# Trends and Patterns of Geographic Variation in Opioid Prescribing Practices by State, United States, 2006-2017

**DOI:** 10.1001/jamanetworkopen.2019.0665

**Published:** 2019-03-15

**Authors:** Lyna Z. Schieber, Gery P. Guy, Puja Seth, Randall Young, Christine L. Mattson, Christina A. Mikosz, Richard A. Schieber

**Affiliations:** 1 Division of Unintentional Injury Prevention, National Center for Injury Prevention and Control, Centers for Disease Control and Prevention, Atlanta, Georgia; 2 Division of Toxicology and Human Health Sciences, Agency for Toxic Substances and Disease Registry, Atlanta, Georgia; 3 Division of Public Health Information and Dissemination, Center for Surveillance, Epidemiology, and Laboratory Sciences, Centers for Disease Control and Prevention, Atlanta, Georgia

## Abstract

**Importance:**

Risk of opioid use disorder, overdose, and death from prescription opioids increases as dosage, duration, and use of extended-release and long-acting formulations increase. States are well suited to respond to the opioid crisis through legislation, regulations, enforcement, surveillance, and other interventions.

**Objective:**

To estimate temporal trends and geographic variations in 6 key opioid prescribing measures in 50 US states and the District of Columbia.

**Design, Setting, and Participants:**

Population-based cross-sectional analysis of opioid prescriptions filled nationwide at US retail pharmacies between January 1, 2006, and December 31, 2017. Data were obtained from the IQVIA Xponent database. All US residents who had an opioid prescription filled at a US retail pharmacy were included.

**Main Outcomes and Measures:**

Primary outcomes were annual amount of opioids prescribed in morphine milligram equivalents (MME) per person; mean duration per prescription in days; and 4 separate prescribing rates—for prescriptions 3 or fewer days, those 30 days or longer, those with a high daily dosage (≥90 MME), and those with extended-release and long-acting formulations.

**Results:**

Between 2006 and 2017, an estimated 233.7 million opioid prescriptions were filled in retail pharmacies in the United States each year. For all states combined, 4 measures decreased: (1) mean (SD) amount of opioids prescribed (mean [SD] decrease, 12.8% [12.6%]) from 628.4 (178.0) to 543.4 (158.6) MME per person, a statistically significant decrease in 23 states; (2) high daily dosage (mean [SD] decrease, 53.1% [13.6%]) from 12.3 (3.4) to 5.6 (1.7) per 100 persons, a statistically significant decrease in 49 states; (3) short-term (≤3 days) duration (mean [SD] decrease, 43.1% [9.4%]) from 18.0 (5.4) to 10.0 (2.5) per 100 persons, a statistically significant decrease in 48 states; and (4) extended-release and long-acting formulations (mean [SD] decrease, 14.7% [13.7%]) from 7.2 (1.9) to 6.0 (1.7) per 100 persons, a statistically significant decrease in 27 states. Two measures increased, each associated with the duration of prescription dispensed: (1) mean (SD) prescription duration (mean [SD] increase, 37.6% [6.9%]) from 13.0 (1.2) to 17.9 (1.4) days, a statistically significant increase in every state; and (2) prescriptions for a term of 30 days or longer (mean [SD] increase, 37.7% [28.9%]) from 18.3 (7.7) to 24.9 (10.7) per 100 persons, a statistically significant increase in 39 states. Two- to 3-fold geographic differences were observed across states, measured by comparing the ratio of each state’s 90th to 10th percentile for each measure.

**Conclusions and Relevance:**

In this study, across 12 years, the mean duration and prescribing rate for long-term prescriptions of opioids increased, whereas the amount of opioids prescribed per person and prescribing rate for high-dosage prescriptions, short-term prescriptions, and extended-release and long-acting formulations decreased. Some decreases were significant, but results were still high. Two- to 3-fold state variation in 5 measures occurred in most states. This information may help when state-specific intervention programs are being designed.

## Key Points

**Question:**

How have key opioid prescription measures changed by state between 2006 and 2017 in the United States, and are changes evenly distributed across states?

**Findings:**

In this cross-sectional study of an estimated 223.7 million retail opioid prescriptions filled annually between 2006 and 2017, the amount of opioids prescribed increased up to 2010, then decreased, for a net reduction of 13%, with the greatest decrease occurring in 2017. One in 3 opioids were prescribed for a duration of 30 days or more, increasing 3% annually; in 5 of 6 measures studied, there was a 2- to-3-fold variation among states.

**Meaning:**

The amount of opioids prescribed decreased, but long-term prescriptions increased, and considerable variation among states existed.

## Teaser

This population-based cross-sectional study uses data from the IQVIA Xponent database for all opioid prescriptions filled in the United States from 2006 through 2017 to evaluate national and state trends in amount, dosage, duration, and formulation.

## Introduction

The recent decline in US life expectancy is attributed, in part, to premature deaths from opioid overdose.^1^ Prescription opioids were involved in approximately 36% of all deaths in the United States associated with opioid overdose in 2017.^2^ The risk of opioid use disorder (commonly called *addiction*), overdose, and death increases as prescription opioids are taken in higher dosages,^3,4,5^ for longer periods of time,^6,7,8^ or as extended-release and long-acting formulations.^9,10,11,12^ Duration of use is the strongest predictor of opioid use disorder and overdose. Each additional week of use has been associated with a 20% increased risk for the development of an opioid use disorder or occurrence of an overdose.^6,8^ Dosage is also important. Overdose risk is dosage dependent, doubling from 50 to 99 morphine milligram equivalents (MME) per day and increasing up to 9-fold at dosages of 100 MME or greater per day compared with overdose risk at dosages less than or equal to 20 MME/d.^3,4,5^ Use of extended-release and long-acting agents also increases risk; unintentional overdoses are twice as likely to occur in those initiating therapy with extended-release and long-acting formulations compared with those starting with immediate-release opioids, especially in the first 2 weeks of use.^10,12^ Prescribing opioids in excess of the patient’s needs may harm the patient as well as others if unused pills are diverted to street sales or misused.^13^ Misuse refers to drugs taken for a purpose other than that directed by the prescribing physician, in greater amounts, more often, or for a longer duration than prescribed. Although the epidemic is shifting from prescription opioids toward street drugs (heroin, fentanyl, and fentanyl analogues), 66% to 83% of new users of heroin report that their addiction began with the misuse of a prescription opioid.^14,15^

Large variation among states has been observed in opioid-related overdose rates and consequent emergency department visits, hospital use, and deaths.^2,16,17^ Long-term trend data on opioid prescribing have been published at the county but not the state level.^18^ However, the states have jurisdictional responsibility to establish and fund state- and often county-level intervention programs, as well as to change state policies, licensing, regulations, legislation, medical reimbursements, surveillance, and professional education concerning prescriptions written. Accordingly, we examined key measures of opioid prescriptions filled in each state from 2006 through 2017, to help guide the development of state-specific interventions.

## Methods

### Data Source

We abstracted data obtained from outpatient prescribing records from the IQVIA Xponent database^19^ from January 1, 2006, through December 31, 2017. This administrative database provides weighted estimates of the number of opioid prescriptions dispensed from approximately 59 400 retail, nonhospital pharmacies that dispense 92% of all retail prescriptions in the United States. National and state estimates of opioid prescriptions filled were computed using these data from each of the 50 states and the District of Columbia, expressed here for simplicity as “51 states.” Data on prescriptions obtained by mail order and those dispensed directly by clinicians or methadone maintenance treatment programs were not available. We excluded cough and cold formulations containing opioids and buprenorphine products commonly used to treat opioid use disorder.^18^ Because records contained no identifying information, this study was determined to be exempt from review by the Centers for Disease Control and Prevention Institutional Review Board. This study followed the Strengthening the Reporting of Observational Studies in Epidemiology (STROBE) reporting guideline for cross-sectional studies.^20^

### Definitions and Variables

Six measures are described at the national and state level: (1) milligrams of a prescribed opioid weighted to the US or state populations, expressed as MME, based on its analgesic potency relative to morphine^21^; (2) mean annual duration per prescription (in days); and (3) 4 weighted annual prescribing rates per 100 persons, namely, opioid prescriptions filled in high dosages (≥90 MME/d), prescriptions filled for 3 days or fewer or 30 days or longer, and prescriptions filled as extended-release and long-acting formulations. The annual amount of opioids prescribed in MME per person and 4 annual prescribing rates were calculated as population-based rates by weighting raw values to national and state populations of each study year.^22,23^ The exception was the mean duration per prescription, which was unweighted.

We defined high-dosage prescriptions as those filled for 90 or more MME/d, above which the risk of opioid use disorder, overdose, and death are greatly increased.^3,4,5,24^ Duration (days of supply filled per prescription) was classified as short-term if 3 or fewer days, which is often sufficient to treat acute pain, or as long-term if 30 or more days, which is more likely used to treat chronic pain.^3^ A 30-day span likely coincides with refills occurring at monthly checkups.^3,25^ Although others define long-term opioid therapy as 90 or more days’ use, 0.3% of all single prescriptions in 2017 noted herein exceeded 89 days’ duration (data not shown).^24^ Prescription formulation was categorized as (1) an immediate-release opioid (eg, oxycodone hydrochloride or terephthalate, oxymorphone hydrochloride, hydrocodone bitartrate, or morphine sulfate) or (2) an extended-release and long-acting opioid, such as either methadone hydrochloride or transdermal fentanyl citrate or as an extended-release and long-acting formulation of an immediate-release drug.

### Statistical Analysis

Data were abstracted and descriptive analyses completed using SAS, version 9.3 (SAS Institute Inc). We report 1-year values for 2006 and 2017, relative percentage change between point estimates for 2006 and 2017, and 12-year and shorter temporal trends of national and state averages of each variable using Joinpoint regression analysis (version 4.5.0.1; National Cancer Institute).^26^ Joinpoint uses log-linear regression to fit the simplest trend of the data and calculate percentage changes. Trends spanning 2006 through 2017 were computed as the mean annual percentage change. Trends of shorter time segments were computed as the annual percentage change. Annual percentage change and mean annual percentage change for each variable are expressed as the percentage change with 95% CI. The terms *increase* and *decrease* refer to an annual percentage change significantly different from zero. All hypothesis testing was 2-tailed, with statistical significance set at 2-sided *P* < .05.

State-level geographic inequality in opioid-prescribing attributes was quantified by comparing the 10th and 90th percentiles for each variable among all states.^27^ The difference between these 2 percentiles was used to indicate the degree of absolute geographic inequality, representing the absolute magnitude of the gap between high- and low-prescribing states for each variable. Use of the 90th percentile instead of the maximum value and the 10th percentile instead of the minimum value reduced the ability of outliers to skew results for each variable. The ratio between the 90th to 10th percentiles was used to assess the relative degree of geographic inequality between states for each variable.

## Results

Each year between 2006 and 2017, an estimated 233.7 million opioid prescriptions (211.0 billion MME) met the inclusion criteria and were filled in retail pharmacies in the United States (Table 1). In 2017, pharmacies filled enough opioid prescriptions to theoretically provide every US resident with 5 mg of hydrocodone bitartrate every 4 hours around the clock for 3 weeks.^28^

**Table 1.** Annual Opioid Prescribing for Key Measures, United States, 2006-2017^a^

Year	Total Opioid Prescriptions, No.	Total Amount of Opioids Prescribed, MME per Person	Prescription Duration			High-Dosage Prescription, %^b^^,^^c^	Prescription for ER/LA Formulation, %^b^
			Mean (Min, Max) [Median], d	≤3 d, %^b^	≥30 d, %^b^		
Total	2 804 913 925	2 531 829 556 808	16.0 (1, 365) [13.5]	18.1	33.5	11.9	9.2
2006	215 917 091	178 983 433 016	13.3 (1, 365) [8.0]	22.5	24.4	15.8	8.8
2007	228 543 586	197 003 023 046	13.9 (1, 365) [9.0]	21.4	26.5	15.4	9.2
2008	237 860 047	212 692 301 908	14.5 (1, 365) [10.0]	20.1	25.4	15.0	9.4
2009	243 741 861	224 904 429 806	15.0 (1, 365) [10.0]	19.4	30.1	14.4	9.3
2010	251 095 243	242 023 173 212	15.5 (1, 365) [10.0]	18.5	31.9	14.0	9.3
2011	252 175 391	238 771 213 485	16.0 (1, 365) [13.0]	18.0	33.5	10.8	9.1
2012	255 215 911	232 647 571 180	16.4 (1, 365) [14.0]	17.5	34.5	10.2	8.8
2013	247 097 560	222 827 081 984	16.9 (1, 365) [15.0]	16.7	36.2	9.8	9.0
2014	240 993 021	215 925 435 233	17.2 (1, 365) [15.0]	16.0	37.6	9.4	9.1
2015	226 819 924	205 835 493 929	17.7 (1, 365) [15.0]	15.7	39.9	9.4	9.5
2016	214 236 023	193 237 685 189	18.1 (1, 365) [20.0]	15.4	41.2	9.2	9.5
2017	191 218 266	166 978 714 820	18.3 (1, 365) [20.0]	15.2	42.0	8.5	9.1
Mean (SD)^d^	233 742 827 (19 143 892)	210 985 796 401 (23 478 521 617)	16.1 (1.7)	18.1 (2.4)	33.6 (6.1)	11.8 (2.8)	9.2 (0.2)

Abbreviations: ER/LA, extended-release and long-acting; Max, maximum; Min, minimum; MME, morphine milligram equivalents.

^a^ Data were obtained from the IQVIA Xponent database.^19^

^b^ Percentage of the total number of opioid prescriptions.

^c^ A high-dosage prescription was defined as having a daily dosage of 90 MME or more.

^d^ The SDs were calculated from single-year values across 12 years.

### Annual Amount of Opioids Filled per Person

**Figure 1.**
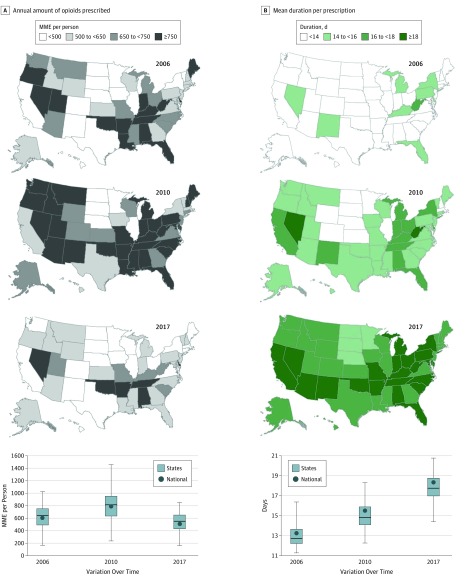
Changes in Annual Amount of Opioids Prescribed in Morphine Milligram Equivalents (MME) per Person, and Mean Duration per Prescription From Years 2006, 2010, and 2017 Data were calculated from the IQVIA Xponent database^19^ in the years 2006, 2010, and 2017. The annual amount of opioids prescribed in MME per person and mean duration per prescription were calculated from all opioids prescribed for each state and the District of Columbia in that year. We used 2010 quartiles as the break points for part A and the break points that optimize the visual differences among states between maps for part B. The dark color indicates a higher MME per person or a longer duration. In the boxplots, the bottom border of the boxes indicates the 25th percentile; middle line, the 50th percentile; top border, the 75th percentile across all states; whiskers, the full range across states; and circles, the national mean for MME per person (A) and mean duration per prescription (B).

Single-year values for the amount of opioids filled (MME per person) varied widely by state and year (Figure 1A, Table 2, and eTable 1 in the Supplement). In 2017, the mean MME per person by state had a large absolute geographic inequality gap of 357.6 MME per person between the 90th percentile and 10th percentile values (Table 2). The ratio of the 90th and 10th percentile state values was 2.2 in 2017, representing approximately a 2-fold variation among states (Table 2). Across 12 years, MME per person decreased by a mean (SD) of 12.8% (12.6%) among states, yet the absolute geographic inequality gap increased by 6.9% and the relative geographic inequality value increased by 17.3% (Table 2). States with the greatest MME per person in 2017 were Tennessee, Oklahoma, Delaware, and Alabama, each exceeding 820 MME per person (eTable 2 in the Supplement).

**Table 2.** Summary of Trends in Prescribing Characteristics for 50 States and the District of Columbia, United States, 2006-2017^a^

Prescribing Characteristic by Year	Mean (SD) [Median]^b^	Percentile		Geographic Inequality		States With Statistically Significant Change	
		10th	90th	Absolute^c^	Relative^d^	Decrease^e^	Increase^f^
Total amount of opioids prescribed, MME per person							
2006	628.4 (178.0) [640.6]	439.9	850.7	410.8	1.9		
2017	543.4 (158.6) [547.1]	357.6	796.8	439.2	2.2		
Change (2006-2017), %	−12.8 (12.6) [−12.1]	−18.7	−6.3	6.9	17.3		
Trend (2006-2017), No. (%)^g^						23 (45.1)	2 (3.9)
Mean duration per prescription, d							
2006	13.0 (1.2) [12.7]	11.6	14.8	3.2	1.3		
2017	17.9 (1.4) [17.7]	16.3	19.7	3.4	1.2		
Change (2006-2017), %	37.6 (6.9) [38.2]	39.9	32.7	6.7	−5.1		
Trend (2006-2017), No. (%)^g^						0	51 (100.0)
Prescription by duration, rate per 100 persons							
≤3 d							
2006	18.0 (5.4) [18.2]	11	24.2	13.2	2.2		
2017	10.0 (2.5) [10.1]	7.0	13.1	6.1	1.9		
Change, %	−43.1 (9.4) [−44.2]	−36.8	−46.1	−53.8	−14.6		
Trend, No. (%)^g^						48 (94.1)	0
≥30 d							
2006	18.3 (7.7) [17.3]	11.1	23.9	12.8	2.2		
2017	24.9 (10.7) [21.1]	13.0	39.5	26.5	3.0		
Change (2006-2017), %	37.7 (28.9) [44.6]	17.0	65.1	106.9	41.1		
Trend (2006-2017), No. (%)^g^						3 (5.8)	39 (76.4)
High-dosage prescription, rate per 100 persons^h^							
2006	12.3 (3.4) [12.7]	8.2	16.3	8.1	2.0		
2017	5.6 (1.7) [5.6]	3.3	7.9	4.6	2.4		
Change (2006-2017), %	−53.1 (13.6) [−56.7]	−59.8	−51.5	−43.2	20.5		
Trend (2006-2017), No. (%)^g^						49 (94.1)	0
Prescription for ER/LA formulation, rate per 100 persons							
2006	7.2 (1.9) [7.3]	4.9	9.7	4.9	2.0		
2017	6.0 (1.7) [6.0]	4.1	8.2	4.1	2.0		
Change (2006-2017), %	−14.7 (13.7) [−12.8]	−15.2	−15.6	−16.3	0.0		
Trend (2006-2017), No. (%)^g^						27 (52.9)	1 (1.9)

Abbreviations: ER/LA, extended-release and long-acting; MME, morphine milligram equivalents.

^a^ Data were obtained from the IQVIA Xponent database.^19^

^b^ Mean was calculated from the values from 50 states and the District of Columbia, expressed here for simplicity as “51 states.” This mean does not reflect the US national value, which can be found in eTables 1 through 6 in the Supplement.

^c^ Measure of absolute geographic inequality was calculated by subtracting the 10th percentile from the 90th percentile.

^d^ Measure of relative geographic inequality was calculated as the ratio of the 90th percentile to the 10th percentile.

^e^ Indicates that a trend was significantly different from zero at the α = .05 level (*P* < .05) and that the mean annual percentage change had a negative value according to joinpoint regression analysis.

^f^ Indicates that a trend was significantly different from zero at the α = .05 level (*P* < .05) and that the mean annual percentage change had a positive value according to joinpoint regression analysis.

^g^ Trend detail for each state can be found in eTables 1 through 6 in the Supplement.

^h^ A high-dosage prescription was defined as having a daily dosage of 90 MME or more.

Joinpoint analysis indicated that MME per person nationally increased annually by 6.9% (95% CI, 5.5%-8.3%; *P* < .001) from 2006 to 2010, then decreased by 3.8% (95% CI, 2.5%-5.0%; *P* < .001) from 2010 through 2015 and by 10.7% (95% CI, 6.6%-14.7%; *P* < .001) from 2015 through 2017 (eTable 1 in the Supplement). By 2016, the overall mean (SD) per-person amount had steadily decreased to a level nearly identical to that in 2006. In 2017, this decreased further, with the only absolute decline evident in a mean (SD) of 14.8% (3.9%) decrease occurring between 2016 and 2017. The net mean (SD) reduction was 12.8% (12.6%) from 2006 to 2017, with the greatest decrease occurring in 2017 (eTable 1 in the Supplement). This pattern was observed in all states except Vermont, the only state where a steady 12-year increase occurred: 2.0% annually (95% CI, 0.9%-3.2%; *P* < .001) (eTable 1 in the Supplement). Across these 12 years, the MME per person in 44 states decreased below the 2006 baseline by a mean (SD) of 12.8% (12.6%) from 628.4 to 543.4 (a statistically significant decline in 23 states) (Table 2 and eTable 1 in the Supplement). The largest declines across the 12-year period were seen in Maine (41.1% overall; 4.3% annually [95% CI, 2.8%-5.7%]; *P* < .001), Massachusetts (36.3% overall; 4.1% annually [95% CI, 2.5%-5.8% ]; *P* < .001), and North Dakota (33.8% overall; 3.7% annually [95% CI, 1.9%-5.5%]; *P* < .001) (eTable 1 in the Supplement).

### Duration per Prescription

The mean prescription duration increased substantially over the 12 years and varied little among states (Figure 1B and Table 2). In 2017, the absolute geographic inequality gap was 3.4 days, with a relative gap ratio of 1.2 (Table 2). States with the highest mean durations in 2017 were Kentucky, Nevada, and West Virginia, each exceeding 20 days (eTable 2 in the Supplement).

Joinpoint analysis indicated that prescription duration nationally increased steadily by 2.9% annually (95% CI, 2.7%-3.2%; *P* < .001), an overall increase of 37.8% from 2006 to 2017 (eTable 2 in the Supplement). This pattern was observed in every state (eTable 2 in the Supplement). Change in duration increased by a mean (SD) of 37.9% (6.9%), from 13.0 (1.2) days in 2006 to 17.9 (1.4) days in 2017 among states (Table 2). The greatest increases over the 12-year period were seen in Oklahoma (54.8% overall; 4.1% annually [95% CI, 3.9%-4.3%]; *P* < .001), New Hampshire (52.5% overall; 3.7% annually [95% CI, 3.5%-3.9%]; *P* < .001), and Idaho (51.2% overall; 3.7% annually [95% CI, 3.3%-4.1%]; *P* < .001) (eTable 2 in the Supplement).

### Prescriptions for Duration of 3 or Fewer Days 

**Figure 2.**
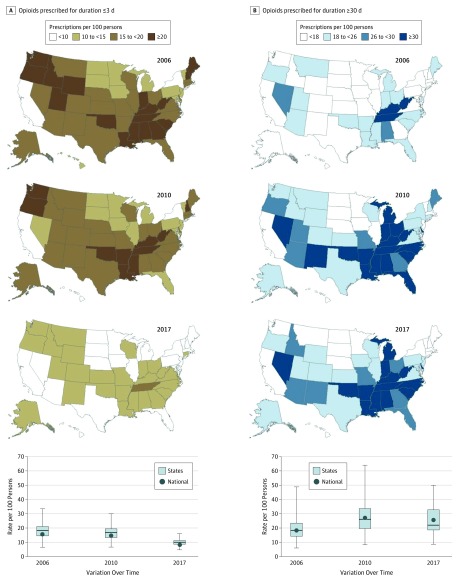
Changes in Rates per 100 Persons of Opioids Prescribed of Duration of 3 or Fewer Days and 30 or More Days From Years 2006, 2010, and 2017. Data were calculated from the IQVIA Xponent database^19^ for the years 2006, 2010, and 2017. Rates of opioids prescribed for a duration of 3 or fewer days (A) and for 30 days or longer (B) were determined from all opioids prescribed for each state and the District of Columbia in that year. The 2010 quartiles were used as the break points for both parts. Dark colors indicate the higher-risk prescribing practices—higher prescribing rate of opioids for a duration of 30 days or longer or 3 or fewer days. In the boxplots, the bottom border of the boxes indicates the 25th percentile; middle line, the 50th percentile; top border, the 75th percentile across all states; whiskers, the full range across states; and circles, the national mean for rates per 100 persons of opioids prescribed for a duration of 3 or fewer days (A) and 30 days or longer (B).

Across these 12 years, a mean (SD) annual 18.1% (2.4%) of prescriptions were filled for a duration of 3 or fewer days (Table 1). Single-year values for annual prescription duration of 3 or fewer days, an attribute with lower risk, varied widely among states (Figure 2A). For example, in 2006, prescription duration of 3 or fewer days had a large absolute geographic inequality of 13.2 per 100 persons and a relative geographic inequality ratio of 2.2 among states (Table 2). However, these disparities among states decreased substantially over these 12 years, in both their absolute geographic inequality gap (53.8%) and relative geographic inequality (14.6%) (Table 2). In 2017, Tennessee, Louisiana, and Mississippi had the highest rate of short-duration prescriptions filled, each more than 14.0 per 100 persons (eTable 3 in the Supplement).

Joinpoint analysis indicated a continuous downward trend in short-term prescriptions from 2006 to 2017 nationally, with an overall relative percentage decline of 45.2%, 5.2% annually (95% CI, 4.7%-5.7%; *P* < .001) (eTable 3 in the Supplement). This pattern was observed with a 12-year mean (SD) state decline of 43.1% (9.4%), which was statistically significant in all states except in the District of Columbia, Iowa, and South Dakota (Table 2 and eTable 3 in the Supplement). The largest declines over the 12-year period were seen in Maine (61.5% overall; 7.6% annually [95% CI, 5.7%-9.5%]; *P* < .001), Oklahoma (60.2% overall; 8.4% annually [95% CI, 7.7%-9.1%]; *P* < .001), and New Hampshire (55.6% overall; 7.0% annually [95% CI, 5.4%-8.6%]; *P* < .001) (eTable 3 in the Supplement).

### Prescriptions for Duration of 30 or More Days

Over these 12 years, a mean (SD) annual 33.6% (6.1%) of opioid prescriptions were filled for 30 or more days (Table 1). Single-year values for prescriptions for 30 or more days varied widely among states (Figure 2B). In 2017, prescriptions for 30 or more days had a large absolute geographic inequality gap of 26.5 per 100 persons and a relative geographic inequality ratio of 3.0 among states (Table 1). Across the 12 years, disparities among states increased substantially, with a marked increase in both absolute geographic inequality gap (106.9%) and relative geographic inequality (41.1%) between 2006 and 2017. Alabama, Tennessee, Kentucky, and Arkansas had the highest rates of prescriptions for 30 or more days filled, each more than 45.0 per 100 persons in 2017 (eTable 4 in the Supplement).

Joinpoint analysis indicated an upward national trend in the prescribing rate of prescriptions for a duration of 30 or more days, with an overall increase of 40.2%, 3.0% annually (95% CI, 2.0%-4.1%; *P* < .001), from 2006 through 2017 (eTable 4 in the Supplement). The pattern of trends varied among states. Across these 12 years, the prescribing rate for a duration of 30 or more days increased a mean (SD) of 37.7% (28.9%) among states, which was a statistically significant increase in 39 states and decrease in 3 states (Table 2 and eTable 4 in the Supplement). The largest increases across the 12-year period were seen in Arkansas (97.4% overall; 6.4% annually [95% CI, 3.7%-9.2%]; *P* < .001), Illinois (82.3% overall; 5.5% annually [95% CI, 4.3%-6.8%]; *P* < .001), and Idaho (79.2% overall; 5.4% annually [95% CI, 4.8%-6.0%]; *P* < .001) (eTable 4 in the Supplement).

### High-Dosage Prescriptions

**Figure 3.**
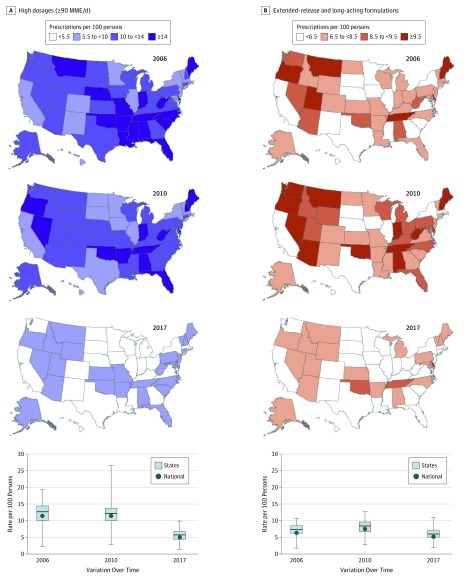
Changes in Rates per 100 Persons of Opioids Prescribed of High Dosages (≥90 MME/d) and Extended-Release and Long-Acting Formulations From Years 2006, 2010, and 2017 Data were calculated from the IQVIA Xponent database^19^ for the years 2006, 2010, and 2017. Rates of opioids prescribed in high dosages (A) and as extended-release and long-acting formulations (B) were determined from all opioids prescribed for each state and the District of Columbia in that year. We used the break points that optimize the visual differences among states between maps for part A and 2010 quartiles as the break points for part B. The darker colors indicate higher-risk prescribing practice—higher prescribing rates of opioids in high dosages or as extended-release and long-acting formulations. In the boxplots, the circles indicate the national mean for rates per 100 persons of opioids prescribed in high dosages (A) and as extended-release and long-acting formulations (B). See the caption to Figure 2 for definitions of the other elements.

Across these 12 years, a mean (SD) annual 11.8% (2.8%) of prescriptions were filled in a high daily dosage (≥90 MME) (Table 1). High-dosage prescribing rates for 2006, 2010, and 2017 each varied widely among states (Figure 3A). In 2017, high-dosage prescribing rates among all states had a large absolute geographic inequality gap of 4.6 per 100 persons and a relative geographic inequality ratio of 2.4 (Table 2). In 2017, Delaware, Utah, and Alaska had the highest prescribing rates, exceeding 8.2 high-dosage prescriptions filled per 100 persons (eTable 5 in the Supplement).

Joinpoint analysis indicated a 56.7% decline in high-dosage prescribing rates nationally, 7.6% annually (95% CI, 7.2%-8.1%; *P* < .001) from 11.5 in 2006 to 5.0 per 100 persons in 2017 (eTable 5 in the Supplement). For these 12 years, the overall trend decreased by a mean (SD) of 53.1% (13.6%) in all states (Table 2) and was statistically significant in all but Hawaii and Vermont (eTable 5 in the Supplement). The magnitude of decrease varied widely among states over this period, with the largest decrease in Texas (76.0% overall; 12.2% annual [95% CI, 10.7%-13.7%]; *P* < .001), North Dakota (74.3% overall; 11.9% annually [95% CI, 10.1%-13.6%]; *P* < .001), and Nebraska (71.6% overall; 11.9% annually [95% CI, 9.7%-14.0%]; *P* < .001) (eTable 5 in the Supplement).

### Prescriptions With Extended-Release and Long-Acting Formulations

Across these 12 years, a mean (SD) annual 9.2% (0.2%) of prescriptions were filled as an extended-release and long-acting formulations (Table 1). Single-year values for the annual prescribing rates for extended-release and long-acting formulations varied widely among states (Figure 3B). In 2017, prescribing rates for extended-release and long-acting formulations had a large absolute geographic inequality gap of 4.1 per 100 persons and a relative geographic inequality ratio of 2.0 among states (Table 2). Delaware, Oklahoma, and Tennessee had the highest prescribing rates of these formulations, with each state exceeding 8.6 per 100 persons (eTable 6 in the Supplement).

Joinpoint analysis indicated an upward trend in prescribing rates for extended-release and long-acting formulations from 2006 to its peak year between 2008 and 2012, depending on the state. This was followed by a downward trend that extended through 2017. Across 12 years, the trend declined by a mean (SD) of 14.7% (13.7%) in 44 states, which was statistically significant for 27 states (Table 2 and eTable 6 in the Supplement). Vermont was the only state with an annual increase: 1.3% (95% CI, 0%-2.6%; *P* < .001) (eTable 6 in the Supplement).

Prescriptions for extended-release and long-acting formulations filled in 2017 had both a high daily dosage (mean [SD], 102.2 [9.7] MME/d) and long duration (mean [SD], 27.8 [0.9] days) in every state (eFigure in the Supplement). On average, 49.5% of all high-dosage prescriptions in 2017 were filled for extended-release and long-acting formulations (data not shown).

Details on trends in all 6 key prescribing measures for each state and their national values can be found in eTables 1 through 6 in the Supplement.

## Discussion

This study documents state and national trends in several key measures of opioid prescribing. The annual mean amount of opioids filled per person for states first rose and then fell back to 2006 levels, with the only absolute decline evident in a 13% decrease occurring between 2016 and 2017. During these 12 years, prescription duration increased in all states, averaging approximately 18 days; nearly 1 in 5 (18.1%) prescriptions were filled for a short term of 3 or fewer days, decreasing by 5.2% annually; approximately 1 in 3 prescriptions (33.6%) were filled for 30 or more days, increasing 3% annually; and high-dose prescriptions decreased by 53%, but half of these were still filled as extended-release and long-acting formulations.

Disparities in these measures among states were apparent. The state 90th percentile value of prescriptions was 3 times that of the 10th percentile for duration of 30 or more days and approximately 2 times that of the 10th percentile for high dosage, duration of 3 or fewer days, amount supplied per person, and use of extended-release and long-acting medications. Only mean duration had a lower ratio. For mean MME per person, this relative geographic inequality increased by 17.3% during this period.

These data provide state programs with their profile for these indicators. State health officials can judge the relative severity of any excess dosage, duration, and/or use of long-term formulations in their jurisdiction. These data may indicate high-potential areas for opioid use prevention and intervention, whether by program interventions, regulations, state-based reimbursement systems, required opioid education for prescribers and pharmacists, enhanced prescription drug monitoring programs (now used in all states), or other means.^29^ State legislation may also be effective. Since 2010, 11 states have passed laws governing the operation of pain-management clinics.^30^ By April 2018, 28 states had adopted guidelines and requirements for prescribers, mostly related to prescribing limits on dosage or duration.^31^ The introduction of state prescription drug–monitoring programs and pain clinic laws have reduced the amount of opioids prescribed by an estimated 6% to 24% in applicable states and reduced prescription opioid overdose death rate by 12%.^32,33,34^ In Florida, where multiple interventions targeted excessive opioid prescribing from 2010 to 2012 (eg, pain clinic regulation and mandated prescription drug–monitoring program reporting of dispensed prescriptions), the present study and others indicate a 53% decline in amounts of opioids prescribed, a 66% decline in high-dosage prescriptions, and fewer prescription opioid–related overdose deaths from 2010 to 2017.^35,36^ Additional studies using rigorous methods are needed to determine the association between opioid use and changes in laws, regulations, and practices.

This study raises several issues. Because duration of use is the factor most often associated with opioid use disorder and overdose,^6,7,8^ the increase in mean duration per prescription and prescribing rate for 30 or more days is notable and worth further investigation. However, the decline in short-duration prescriptions may indicate a growing awareness by prescribers that nonopioid medications and other pain-control measures, such as exercise and cognitive behavioral therapy, can be effective for short-term pain relief.^37,38^ Physician surveys or medical record reviews could help assess this effectiveness. If that is the case, some risk of early-onset opioid use disorder from prescription opioids may be lessened.

Although the number of opioid-related deaths from all sources increased since 2012, the number of deaths each year associated with use of prescription opioids alone has not increased since then.^16,39,40^ This may be partly owing to an overall decline in the amount of opioids prescribed. Meanwhile, the relatively recent rise in opioid-related deaths may be owing to greater use of illicit drugs, especially if they are cheaper than prescription opioids.^15,41^ Death may then result from illicit drug use by itself, or tainted by fentanyl or its analogues, or combined with prescription opioids.^42^ Accordingly, both illicit street drugs and prescription opioids must become less available. This highlights the complexity of solving the current epidemic. Closing the path to opioid use disorder will require addressing overprescription of legal opioids, reducing the availability of illicit opioids, and getting patients with opioid use disorder into treatment.

### Limitations

This study is subject to several limitations. This administrative database contained no clinical information, including the reason opioids were prescribed or continued, nor any longitudinal data linking patients to clinical outcomes. We could not link the patient’s and physician’s personal identification, demographic information, or physician specialty. Data were not age adjusted. The number of prescriptions filled may be smaller than the number prescribed if prescriptions were not filled. Cutoff values of 3 or fewer days, 30 or more days, and 90 or more MME per day were applied to all patients without knowledge of individual needs or their total duration of opioid therapy. Because prescriptions were anonymized, it is impossible to identify patients receiving multiple simultaneous prescriptions, although each patient received a mean of 3.4 prescriptions in 2017.^43^ We acknowledge that, in pain treatment, 1 size does not fit all, and different approaches and cutoff values may be needed for some groups of individuals.^3,4,5,24,37,38^ Population-based analysis may not apply to an individual’s prescription or needs.

## Conclusions

These data may be able to inform states as they create laws, policies, communications, and interventions tailored to their specific problems. The increase in prescription duration and prescribing rate of prescriptions for 30 or more days are notable trends. The magnitude, severity, and chronic nature of the opioid epidemic in the United States is of serious concern to clinicians, the government, the general public, and many others. As they review new studies and recommendations, clinicians should continue to consider how they might improve pain management, including opioid prescribing, in their own practice.

## Notes

Supplement. **eTable 1.** Trends in Annual Amount of Opioids Prescribed in Morphine Milligram Equivalents (MME) per Person in All Ages, by State, United States, 2006-2017 **eTable 2.** Trends in Mean Annual Duration per Prescription, by State, United States, 2006-2017 **eTable 3.** Trends in Rate (per 100 Population) of Opioids Prescribed for Duration ≤3 Days, by State, United States, 2006-2017 **eTable 4.** Trends in Rate (per 100 Persons) of Opioids Prescribed for Duration ≥30 Days, by State, United States, 2006-2017 **eTable 5.** Trends in Rate (per 100 Population) of Opioids Prescribed in High Dosages (≥90 MME per Day), by State, United States, 2006-2017 **eTable 6.** Trends in Rate (per 100 Population) of Opioids Prescribed as Extended-Release or Long-Acting, by State, United States, 2006-2017 **eFigure.** (A) Mean Dosage in Morphine Milligram Equivalent (MME) per Day per Prescription and (B) Mean Duration per Prescription by Formulation in All Ages, by State, United States, 2017
