# Errors in eFigure Title and Caption in the Supplement

**DOI:** 10.1001/jamanetworkopen.2019.4307

**Published:** 2019-05-03

In the Original Investigation titled “Trends and Patterns of Geographic Variation in Opioid Prescribing Practices by State, United States, 2006-2017,”^1^ published March 15, 2019, a typographical error appeared in the title of the Supplement’s eFigure, and descriptions of 2 symbols used in the eFigure’s graphs were reversed. The eFigure’s title should have read as follows: “eFigure. (A) Mean Dosage in Morphine Milligram Equivalent (MME) per Day per Prescription and (B) Mean Duration per Prescription by Formulation in All Ages, by State, United States, 2017.” In the caption, the descriptions of what is represented in the graphs by the black triangle and the red square were reversed. The last sentence should have read, “Blue diamond represents extended release and long acting (ER/LA) formulations; black triangle represents immediate release (IR) formulations, and red square represents all opioids.” This article has been corrected.^1^
