# Supplementary material for: Trends and Patterns of Geographic Variation in Opioid Prescribing Practices by State, United States, 2006-2017
Source: JAMA Netw Open. 2019 Mar 15;2(3):e190665. doi: 10.1001/jamanetworkopen.2019.0665 (PMC6484643; doi:10.1001/jamanetworkopen.2019.0665)
Supplement: Supplement. — eTable 1. Trends in Annual Amount of Opioids Prescribed in Morphine Milligram Equivalents (MME) per Person in All Ages, by State, United States, 2006-2017 eTable 2. Trends in Mean Annual Duration per Prescription, by State, United States, 2006-2017 eTable 3. Trends in Rate (per 100 Population) of Opioids Prescribed for Duration ≤3 Days, by State, United States, 2006-2017 eTable 4. Trends in Rate (per 100 Persons) of Opioids Prescribed for Duration ≥30 Days, by State, United States, 2006-2017 eTable 5. Trends in Rate (per 100 Population) of Opioids Prescribed in High Dosages (≥90 MME per Day), by State, United States, 2006-2017 eTable 6. Trends in Rate (per 100 Population) of Opioids Prescribed as Extended-Release or Long-Acting, by State, United States, 2006-2017 eFigure. (A) Mean Dosage in Morphine Milligram Equivalent (MME) per Day per Prescription and (B) Mean Duration per Prescription by Formulation in All Ages, by State, United States, 2017 [file jamanetwopen-2-e190665-s001.pdf]

Supplementary Online Content

Schieber LZ, Guy GP Jr, Seth P, et al. Trends and patterns of geographic variation in opioid prescribing practices by state, United States, 2006-2017. *JAMA Netw Open.* 2019;2(3):e190665. doi:10.1001/jamanetworkopen.2019.0665

eTable 1. Trends in Annual Amount of Opioids Prescribed in Morphine Milligram Equivalents (MME) per Person in All Ages, by State, United States, 2006-2017

eTable 2. Trends in Mean Annual Duration per Prescription, by State, United States, 2006-2017

eTable 3. Trends in Rate (per 100 Population) of Opioids Prescribed for Duration ≤3 Days, by State, United States, 2006-2017

eTable 4. Trends in Rate (per 100 Persons) of Opioids Prescribed for Duration ≥30 Days, by State, United States, 2006-2017

eTable 5. Trends in Rate (per 100 Population) of Opioids Prescribed in High Dosages (≥90 MME per Day), by State, United States, 2006-2017

eTable 6. Trends in Rate (per 100 Population) of Opioids Prescribed as Extended-Release or Long-Acting, by State, United States, 2006-2017

eFigure. (A) Mean Dosage in Morphine Milligram Equivalent (MME) per Day per Prescription and (B) Mean Duration per Prescription by Formulation in All Ages, by State, United States, 2017

This supplementary material has been provided by the authors to give readers additional information about their work.

© 2019 Schieber LZ et al. *JAMA Network Open.*

eTable 1. Trends in Annual Amount of Opioids Prescribed in Morphine Milligram Equivalents (MME) per Person in All Ages, by State, United States, 2006-2017

	MME per Person			Change, %		AAPC (95%CI)	Trend 1		Trend 2		Trend 3	
State	2006	2016	2017	2016-2017	2006 - 2017	2006-2017	Years[a]	APC (95% CI)	Years[a]	APC (95% CI)	Years[a]	APC (95% CI)
**United States**	599.9	598.0	512.6	-14.3	-14.5	-1.4 (-2.1 to -0.6)[b]	2006-2010	6.9 (5.5 to 8.3)[b]	2010-2015	-3.8 (-5.0 to -2.5)[b]	2015-2017	-10.7 (-14.7 to -6.6)[b]
**Alabama**	808.8	981.9	828.1	-15.7	2.4	0.4 (-1.5 to 2.3)	2006-2010	7.5 (3.8 to 11.3)[b]	2010-2014	1.1 (-3.8 to 6.3)	2014-2017	-9.3 (-14.1 to -4.2)[b]
**Alaska**	614.4	646.9	576.5	-10.9	-6.2	0.0 (-1.3 to 1.4)	2006-2009	5.5 (0.1 to 11.3)[b]	2009-2017	-2.0 (-3.1 to -0.8)[b]		
**Arizona**	735.0	783.9	647.3	-17.4	-11.9	-1.0 (-2.2 to 0.2)	2006-2011	7.0 (5.5 to 8.5)[b]	2011-2015	-4.4 (-7.1 to -1.6)[b]	2015-2017	-12.5 (-18.1 to -6.4)[b]
**Arkansas**	765.7	901.6	796.8	-11.6	4.1	0.9 (-1.6 to 3.3)	2006-2009	8.6 (-1.1 to 19.2)	2009-2017	-1.9 (-3.8 to 0)		
**California**	450.2	421.6	357.6	-15.2	-20.6	-2.0 (-3.2 to -0.8)[b]	2006-2010	6.4 (4.1 to 8.7)[b]	2010-2014	-2.9 (-6 to 0.3)	2014-2017	-11.2 (-14.5 to -7.7)[b]
**Colorado**	495.4	516.2	433.7	-16.0	-12.4	-1.2 (-2.2 to -0.2)[b]	2006-2011	8.3 (7.1 to 9.5)[b]	2011-2015	-6.0 (-8.2 to -3.8)[b]	2015-2017	-13.3 (-18.0 to -8.4)[b]
**Connecticut**	648.3	569.7	471.6	-17.2	-27.3	-2.9 (-4.3 to -1.5)[b]	2006-2012	2.7 (1.7 to 3.7)[b]	2012-2015	-4.9 (-10.1 to 0.7)	2015-2017	-15.1 (-20.6 to -9.2)[b]
**Delaware**	881.5	974.2	828.2	-15.0	-6.1	-1.0 (-2.2 to 0.2)	2006-2011	9.9 (7.4 to 12.6)[b]	2011-2017	-9.3 (-11.0 to -7.6)[b]		
**District of Columbia**	170.4	180.8	160.1	-11.5	-6.1	-2.3 (-6.6 to 2.1)	2006-2014	2.9 (-0.6 to 6.6)	2014-2017	-15.1 (-28.7 to 1.1)		
**Florida**	807.7	673.3	616.1	-8.5	-23.7	-2.2 (-3.6 to -0.7)[b]	2006-2010	14.3 (11.9 to 16.7)[b]	2010-2013	-17.0 (-22.2 to -11.5)[b]	2013-2017	-5.3 (-7.7 to -2.8)[b]
**Georgia**	548.3	613.5	563.8	-8.1	2.8	0.4 (-0.3 to 1.1)	2006-2010	6.9 (5.0 to 8.9)[b]	2010-2017	-3.1 (-3.9 to -2.4)[b]		
**Hawaii**	407.5	409.7	359.6	-12.2	-11.8	-0.8 (-2.4 to 0.7)	2006-2010	10.3 (7.7 to 13.0)[b]	2010-2013	-2.3 (-8.7 to 4.5)	2013-2017	-9.8 (-12.1 to -7.6)[b]
**Idaho**	561.1	726.0	645.2	-11.1	15.0	1.3 (0.9 to 1.6)[b]	2006-2011	7.8 (7.4 to 8.3)[b]	2011-2015	-0.8 (-1.7 to 0)	2015-2017	-9.8 (-11.4 to -8.1)[b]
**Illinois**	366.0	410.9	359.9	-12.4	-1.7	-0.2 (-1.6 to 1.2)	2006-2010	4.5 (2.0 to 7.0)[b]	2010-2015	-0.8 (-3.1 to 1.5)	2015-2017	-7.4 (-14.3 to 0.1)
**Indiana**	756.6	725.0	605.6	-16.5	-20.0	-1.9 (-4 to 0.3)	2006-2010	7.2 (3.8 to 10.8)[b]	2010-2013	-1.0 (-9.9 to 8.9)	2013-2017	-10.8 (-14 to -7.6)[b]

	MME per Person			Change, %		AAPC (95%CI)	Trend 1		Trend 2		Trend 3	
State	2006	2016	2017	2016-2017	2006 - 2017	2006-2017	Years[a]	APC (95% CI)	Years[a]	APC (95% CI)	Years [a]	APC (95% CI)
Iowa	443.3	460.6	395.1	-14.2	-10.9	-1.1 (-3 to 0.9)	2006-2012	2.9 (1.5 to 4.4)[b]	2012-2015	-2.3 (-9.9 to 5.8)	2015-2017	-10.6 (-18.1 to -2.3)[b]
Kansas	600.5	682.3	610.0	-10.6	1.6	0.0 (-1.6 to 1.6)	2006-2011	3.9 (2.0 to 5.8)[b]	2011-2015	-0.8 (-4.6 to 3.2)	2015-2017	-7.5 (-15 to 0.6)
Kentucky	850.7	813.6	715.5	-12.1	-15.9	-1.7 (-2.6 to -0.8)[b]	2006-2011	6.0 (4.2 to 7.8)[b]	2011-2017	-7.7 (-8.9 to -6.4)[b]		
Louisiana	769.2	711.8	625.8	-12.1	-18.6	-1.6 (-3.2 to 0)	2006-2008	5.4 (-4.6 to 16.5)	2008-2017	-3.1 (-4 to -2.2)[b]		
Maine	827.8	718.1	487.7	-32.1	-41.1	-4.3 (-5.7 to -2.8)[b]	2006-2010	5.7 (3.3 to 8.2)[b]	2010-2015	-3.8 (-6 to -1.6)[b]	2015-2017	-22.4 (-29.2 to -15.0)[b]
Maryland	558.7	605.0	524.1	-13.4	-6.2	0.0 (-1.1 to 1.1)	2006-2010	10.5 (7.3 to 13.8)[b]	2010-2017	-5.6 (-6.7 to -4.4)[b]		
Massachusetts	539.9	409.5	343.9	-16.0	-36.3	-4.1 (-5.8 to -2.5)[b]	2006-2012	1.5 (0.3 to 2.6)[b]	2012-2015	-7.1 (-13.2 to -0.7)[b]	2015-2017	-15.2 (-21.8 to -8.1)[b]
Michigan	665.9	773.5	649.2	-16.1	-2.5	-0.3 (-1.2 to 0.7)	2006-2010	7.3 (5.5 to 9.1)[b]	2010-2015	-0.9 (-2.4 to 0.6)	2015-2017	-12.3 (-16.9 to -7.4)[b]
Minnesota	349.8	348.2	296.1	-15.0	-15.4	-1.5 (-2.6 to -0.4)[b]	2006-2010	6.5 (4.7 to 8.5)[b]	2010-2015	-2.7 (-4.4 to -1.1)[b]	2015-2017	-13.2 (-18.4 to -7.6)[b]
Mississippi	683.4	743.7	646.1	-13.1	-5.5	-0.5 (-1.9 to 1)	2006-2009	6.0 (1.9 to 10.2)[b]	2009-2015	-0.9 (-2.5 to 0.8)	2015-2017	-8.2 (-15.3 to -0.6)[b]
Missouri	663.6	738.0	651.7	-11.7	-1.8	-0.2 (-1.3 to 0.9)	2006-2010	4.3 (2.3 to 6.2)[b]	2010-2015	-0.9 (-2.7 to 0.9)	2015-2017	-6.9 (-12.4 to -1.1)[b]
Montana	694.3	640.1	515.4	-19.5	-25.8	-2.2 (-4.2 to -0.3)[b]	2006-2008	10.4 (-0.9 to 22.9)	2008-2014	-1.2 (-3.4 to 1.1)	2014-2017	-11.8 (-16.9 to -6.4)[b]
Nebraska	475.8	432.8	385.1	-11.0	-19.1	-2.0 (-3.7 to -0.3)[b]	2006-2015	-0.7 (-1.7 to 0.2)	2015-2017	-7.6 (-17.2 to 3.1)		
Nevada	1019.9	932.5	802.9	-13.9	-21.3	-2.0 (-3.5 to -0.6)[b]	2006-2011	5.7 (2.7 to 8.7)[b]	2011-2017	-8.0 (-10.1 to -6)[b]		
New Hampshire	647.3	657.1	553.3	-15.8	-14.5	-1.2 (-2.1 to -0.4)[b]	2006-2009	7.6 (4.9 to 10.3)[b]	2009-2014	0.8 (-0.6 to 2.3)	2014-2017	-12.5 (-14.7 to -10.2)[b]
New Jersey	594.5	619.2	510.8	-17.5	-14.1	-1.1 (-1.7 to -0.5)[b]	2006-2011	5.2 (4.4 to 5.9)[b]	2011-2015	-3.4 (-4.8 to -1.9)[b]	2015-2017	-11.1 (-14 to -8)[b]
New Mexico	488.9	589.7	483.7	-18.0	-1.1	0.2 (-1.6 to 2)	2006-2011	10.8 (7.0 to 14.7)[b]	2011-2017	-7.9 (-10.3 to -5.4)[b]		
New York	441.6	492.2	415.0	-15.7	-6.0	-0.4 (-1.4 to 0.7)	2006-2010	8.7 (6.7 to 10.7)[b]	2010-2015	-2.7 (-4.4 to -1.0)[b]	2015-2017	-11.2 (-16.5 to -5.6)[b]
North Carolina	712.2	767.7	643.2	-16.2	-9.7	-0.8 (-2.1 to 0.4)	2006-2010	4.3 (2 to 6.6)[b]	2010-2015	-0.4 (-2.5 to 1.7)	2015-2017	-11.3 (-17.6 to -4.6)[b]

State	MME per Person 2006	2016	2017	Change, % 2016-2017	2006 - 2017	AAPC (95%CI) 2006-2017	Trend 1 Years[a]	APC (95% CI)	Trend 2 Years[a]	APC (95% CI)	Trend 3 Years[a]	APC (95% CI)
**North Dakota**	439.9	357.0	291.1	-18.5	-33.8	-3.7 (-5.5 to -1.9)[b]	2006-2008	5.5 (-3.4 to 15.1)	2008-2015	-2.4 (-3.8 to -0.9)[b]	2015-2017	-16.0 (-24.3 to -6.8)[b]
**Ohio**	708.9	582.5	483.4	-17.0	-31.8	-3.4 (-4.0 to -2.8)[b]	2006-2010	6.1 (5.2 to 7.1)[b]	2010-2015	-6.7 (-7.6 to -5.9)[b]	2015-2017	-12.7 (-15.6 to -9.6)[b]
**Oklahoma**	884.9	947.1	838.6	-11.5	-5.2	-0.3 (-1.8 to 1.3)	2006-2010	6.3 (3.8 to 8.8)[b]	2010-2013	0.8 (-5.8 to 7.8)	2013-2017	-7.1 (-9.3 to -4.9)[b]
**Oregon**	773.1	669.7	547.1	-18.3	-29.2	-3.1 (-4.6 to -1.7)[b]	2006-2010	7.3 (4.6 to 10)[b]	2010-2014	-4.3 (-7.9 to -0.5)[b]	2014-2017	-14.1 (-18 to -10)[b]
**Pennsylvania**	640.6	718.7	604.8	-15.9	-5.6	-0.5 (-1.3 to 0.3)	2006-2010	7.4 (5.8 to 8.9)[b]	2010-2015	-1.3 (-2.6 to 0)[b]	2015-2017	-12.6 (-16.7 to -8.4)[b]
**Rhode Island**	593.1	513.2	422.1	-17.7	-28.8	-2.8 (-3.9 to -1.7)[b]	2006-2012	3.1 (1.4 to 4.7)[b]	2012-2017	-9.5 (-11.5 to -7.3)[b]		
**South Carolina**	691.5	771.6	671.3	-13.0	-2.9	-0.4 (-1.6 to 1)	2006-2014	2.6 (1.5 to 3.7)[b]	2014-2017	-7.7 (-12.2 to -3)[b]		
**South Dakota**	385.5	396.4	339.0	-14.5	-12.1	-1.7 (-3.9 to 0.6)	2006-2015	1.1 (-0.1 to 2.3)	2015-2017	-13.1 (-24.7 to 0.2)		
**Tennessee**	938.3	998.6	845.7	-15.3	-9.9	-0.8 (-1.7 to 0.1)	2006-2012	6.8 (5.4 to 8.2)[b]	2012-2017	-9.2 (-10.9 to -7.5)[b]		
**Texas**	453.1	403.3	364.6	-9.6	-19.5	-1.9 (-3 to -0.7)[b]	2006-2010	3.7 (0.6 to 6.9)[b]	2010-2017	-5.0 (-6.2 to -3.6)[b]		
**Utah**	753.1	758.0	657.7	-13.2	-12.7	-1.1 (-2.3 to 0)[b]	2006-2008	8.7 (2.7 to 15.0)[b]	2008-2015	-1.4 (-2.3 to -0.5)[b]	2015-2017	-9.3 (-14.6 to -3.8)[b]
**Vermont**	464.2	606.4	536.3	-11.6	15.5	2.0 (0.9 to 3.2)[b]	2006-2017	2.0 (0.9 to 3.2)[b]				
**Virginia**	506.4	537.1	442.7	-17.6	-12.6	-1.1 (-2.1 to 0)[b]	2006-2010	7.3 (5.4 to 9.2)[b]	2010-2015	-2.3 (-3.9 to -0.6)[b]	2015-2017	-13.2 (-18.2 to -7.8)[b]
**Washington**	656.9	559.8	475.6	-15.0	-27.6	-2.9 (-3.8 to -2.1)[b]	2006-2009	5.4 (3.1 to 7.8)[b]	2009-2015	-3.8 (-4.8 to -2.8)[b]	2015-2017	-11.8 (-16.3 to -7.0)[b]
**West Virginia**	1011.6	849.1	675.1	-20.5	-33.3	-3.3 (-4.3 to -2.2)[b]	2006-2009	8.6 (5.3 to 12.0)[b]	2009-2014	-2.4 (-4.2 to -0.5)[b]	2014-2017	-15.1 (-18.1 to -12)[b]
**Wisconsin**	549.1	574.1	451.7	-21.3	-17.7	-1.8 (-3.2 to -0.3)[b]	2006-2011	5.0 (3.3 to 6.8)[b]	2011-2015	-2.5 (-5.9 to 0.9)	2015-2017	-15.5 (-22.1 to -8.3)[b]
**Wyoming**	555.3	654.5	569.1	-13.1	2.5	0.0 (-1.5 to 1.5)	2006-2015	2.4 (1.6 to 3.2)[b]	2015-2017	-10.2 (-18 to -1.7)[b]		

Source: IQVIA Xponent database.

Abbreviation: AAPC, average annual percent change; APC, annual percent change; MME, morphine milligram equivalents; 95% CI, 95% confidence interval.

[a] Year category presented in each trend represented year groupings as determined by joinpoint regression.

[b] Indicates that the Annual Percent Change (APC) or average APC was significantly different from zero at the alpha = 0.05 level.

eTable 2. Trends in Mean Annual Duration per Prescription, by State, United States, 2006-2017

	Duration per Rx, days		Change, %	AAPC (95% CI)	Trend 1		Trend 2		Trend 3	
**State**	**2006**	**2017**	**2006-2017**	**2006-2017**	**Years[a]**	**APC (95% CI)**	**Years[a]**	**APC (95% CI)**	**Years[a]**	**APC (95% CI)**
**United States**	13.3	18.3	37.8	2.9 (2.7 to 3.2)[b]	2006-2011	3.8 (3.3 to 4.2)[b]	2011-2017	2.3 (2.0 to 2.6)[b]		
**Alabama**	13.6	19.4	43.1	3.2 (3.0 to 3.5)[b]	2006-2010	4.5 (4.1 to 4.9)[b]	2010-2014	3.3 (2.7 to 3.9)[b]	2014-2017	1.5 (1.0 to 2.1)[b]
**Alaska**	13.2	17.7	34.1	2.7 (2.4 to 3.0)[b]	2006-2017	2.7 (2.5 to 2.9)[b]				
**Arizona**	13.5	19.3	42.6	3.4 (3.1 to 3.6)[b]	2006-2013	3.9 (3.6 to 4.2)[b]	2013-2017	2.5 (1.9 to 3.1)[b]		
**Arkansas**	12.9	18.4	42.9	3.1 (2.9 to 3.4)[b]	2006-2017	3.1 (2.9 to 3.4)[b]				
**California**	13.7	18.7	36.2	2.9 (2.6 to 3.2)[b]	2006-2010	4.2 (3.4 to 5.1)[b]	2010-2017	2.1 (1.8 to 2.4)[b]		
**Colorado**	11.9	16.5	38.9	3.0 (2.7 to 3.3)[b]	2006-2010	3.9 (3 to 4.8)[b]	2010-2017	2.5 (2.2 to 2.9)[b]		
**Connecticut**	12.7	16.3	27.8	2.3 (2.1 to 2.4)[b]	2006-2013	2.7 (2.5 to 2.9)[b]	2013-2017	1.5 (1.1 to 1.9)[b]		
**Delaware**	14.4	20.1	39.8	3.0 (2.9 to 3.2)[b]	2006-2011	3.7 (3.3 to 4.0)[b]	2011-2017	2.5 (2.2 to 2.7)[b]		
**District of Columbia**	11.3	14.3	27.0	1.8 (0.8 to 2.8)[b]	2006-2017	1.8 (0.8 to 2.8)[b]				
**Florida**	15.0	19.7	31.0	2.5 (2.3 to 2.8)[b]	2006-2010	4.8 (4.1 to 5.4)[b]	2010-2017	1.3 (1.1 to 1.5)[b]		
**Georgia**	12.4	17.7	43.4	3.3 (3.0 to 3.5)[b]	2006-2011	4.4 (3.9 to 4.9)[b]	2011-2017	2.4 (2.0 to 2.7)[b]		
**Hawaii**	12.3	16.3	32.4	2.5 (2.2 to 2.8)[b]	2006-2010	4.3 (3.6 to 5.1)[b]	2010-2017	1.5 (1.2 to 1.7)[b]		
**Idaho**	11.8	17.9	51.2	3.7 (3.3 to 4.1)[b]	2006-2010	4.8 (4 to 5.5)[b]	2010-2015	3.9 (3.2 to 4.6)[b]	2015-2017	1.8 (-0.1 to 3.8)
**Illinois**	11.6	17.1	47.2	3.6 (3.5 to 3.8)[b]	2006-2017	3.6 (3.5 to 3.8)[b]				
**Indiana**	13.2	18.6	40.8	3.3 (3.0 to 3.6)[b]	2006-2011	5.2 (4.9 to 5.5)[b]	2011-2015	2.5 (2 to 3)[b]	2015-2017	-0.4 (-1.4 to 0.6)
**Iowa**	12.2	16.4	34.4	2.6 (1.7 to 3.6)[b]	2006-2008	6.4 (0.3 to 12.8)[b]	2008-2017	1.8 (1.3 to 2.3)[b]		
**Kansas**	11.7	17.0	44.8	3.4 (3.2 to 3.6)[b]	2006-2017	3.4 (3.2 to 3.6)[b]				

State	Duration per Rx, days 2006	2017	Change, % 2006-2017	AAPC (95% CI) 2006-2017	Trend 1 Years[a]	APC (95% CI)	Trend 2 Years[a]	APC (95% CI)	Trend 3 Years[a]	APC (95% CI)
**Kentucky**	15.2	20.7	35.8	2.7 (2.6 to 2.9)[b]	2006-2009	4.2 (3.8 to 4.7)[b]	2009-2013	3.4 (3.0 to 3.9)[b]	2013-2017	1.1 (0.9 to 1.4)[b]
**Louisiana**	12.1	17.1	41.0	3.2 (3.0 to 3.3)[b]	2006-2017	3.2 (3.0 to 3.3)[b]				
**Maine**	13.6	17.8	31.0	2.3 (1.7 to 2.9)[b]	2006-2015	2.8 (2.4 to 3.1)[b]	2015-2017	0.5 (-3.0 to 4.1)		
**Maryland**	13.2	18.2	38.5	2.9 (2.7 to 3.2)[b]	2006-2009	3.8 (2.7 to 4.8)[b]	2009-2017	2.6 (2.4 to 2.8)[b]		
**Massachusetts**	12.6	17.0	34.7	2.9 (2.7 to 3.1)[b]	2006-2009	2.1 (1.4 to 2.8)[b]	2009-2012	4.0 (2.7 to 5.4)[b]	2012-2017	2.4 (2.1 to 2.7)[b]
**Michigan**	14.8	20.5	38.2	3.0 (2.7 to 3.3)[b]	2006-2010	4.2 (3.7 to 4.7)[b]	2010-2015	2.9 (2.5 to 3.3)[b]	2015-2017	1.0 (-0.3 to 2.3)
**Minnesota**	11.3	15.7	38.6	3.1 (2.9 to 3.3)[b]	2006-2010	3.7 (3.1 to 4.4)[b]	2010-2017	2.7 (2.5 to 3.0)[b]		
**Mississippi**	12.7	17.8	40.3	3.1 (2.9 to 3.4)[b]	2006-2010	4.5 (3.9 to 5.1)[b]	2010-2017	2.3 (2.1 to 2.6)[b]		
**Missouri**	12.6	18.2	44.8	3.4 (3.3 to 3.5)[b]	2006-2017	3.4 (3.3 to 3.5)[b]				
**Montana**	13.3	17.4	30.3	2.6 (2.1 to 3.0)[b]	2006-2008	5.2 (2.4 to 8.0)[b]	2008-2017	2.0 (1.8 to 2.2)[b]		
**Nebraska**	11.5	15.6	36.2	2.8 (2.5 to 3.1)[b]	2006-2012	2.2 (1.8 to 2.6)[b]	2012-2017	3.6 (3.1 to 4.1)[b]		
**Nevada**	15.8	20.8	31.4	2.5 (2.2 to 2.7)[b]	2006-2010	3.6 (2.9 to 4.4)[b]	2010-2017	1.8 (1.5 to 2.1)[b]		
**New Hampshire**	11.6	17.7	52.5	3.7 (3.5 to 3.9)[b]	2006-2017	3.7 (3.5 to 3.9)[b]				
**New Jersey**	13.3	17.9	35.0	2.7 (2.5 to 2.9)[b]	2006-2011	3.2 (2.9 to 3.6)[b]	2011-2017	2.3 (2.0 to 2.5)[b]		
**New Mexico**	14.1	18.6	31.7	2.6 (2.4 to 2.8)[b]	2006-2011	3.9 (3.5 to 4.2)[b]	2011-2017	1.5 (1.3 to 1.8)[b]		
**New York**	15.0	18.8	25.6	2.1 (1.8 to 2.5)[b]	2006-2010	3.3 (2.8 to 3.8)[b]	2010-2015	1.8 (1.4 to 2.3)[b]	2015-2017	0.2 (-1.2 to 1.6)
**North Carolina**	13.7	18.7	36.8	2.8 (2.7 to 2.9)[b]	2006-2017	2.8 (2.7 to 2.9)[b]				
**North Dakota**	12.8	15.8	23.2	1.9 (1.7 to 2.2)[b]	2006-2017	1.9 (1.7 to 2.2)[b]				
**Ohio**	13.9	18.3	31.7	2.5 (2.4 to 2.7)[b]	2006-2010	3.6 (3.1 to 4.1)[b]	2010-2017	1.9 (1.7 to 2.1)[b]		
**Oklahoma**	12.4	19.3	54.8	4.1 (3.9 to 4.3)[b]	2006-2015	4.4 (4.3 to 4.5)[b]	2015-2017	3.0 (1.9 to 4.1)[b]		

State	Duration per Rx, days		Change, %	AAPC (95% CI)	Trend 1		Trend 2		Trend 3	
	2006	2017	2006-2017	2006-2017	Years[a]	APC (95% CI)	Years[a]	APC (95% CI)	Years[a]	APC (95% CI)
**Oregon**	12.9	17.6	36.9	2.8 (2.6 to 3.1)[b]	2006-2009	4.7 (3.5 to 6.0)[b]	2009-2013	2.9 (1.7 to 4.0)[b]	2013-2017	1.4 (0.7 to 2.1)[b]
**Pennsylvania**	14.0	19.1	35.8	2.7 (2.6 to 2.8)[b]	2006-2009	3.4 (2.5 to 4.3)[b]	2009-2012	2.1 (0.5 to 3.8)[b]	2012-2017	2.9 (2.5 to 3.2)[b]
**Rhode Island**	12.6	16.6	31.9	2.6 (2.3 to 2.9)[b]	2006-2013	3.2 (2.9 to 3.5)[b]	2013-2017	1.6 (0.9 to 2.3)[b]		
**South Carolina**	12.7	18.4	44.7	3.4 (3.2 to 3.5)[b]	2006-2012	4.3 (4.0 to 4.5)[b]	2012-2017	2.3 (2.0 to 2.6)[b]		
**South Dakota**	12.0	15.4	28.1	2.3 (1.7 to 2.8)[b]	2006-2017	2.0 (1.6 to 2.4)[b]				
**Tennessee**	13.8	19.2	39.1	3.0 (2.8 to 3.2)[b]	2006-2011	4.6 (4.1 to 5.0)[b]	2011-2014	2.5 (0.8 to 4.2)[b]	2014-2017	1.0 (0.2 to 1.8)[b]
**Texas**	12.3	17.3	41.4	3.1 (2.7 to 3.4)[b]	2006-2015	3.4 (3.2 to 3.6)[b]	2015-2017	1.8 (-0.2 to 3.8)		
**Utah**	13.0	17.6	35.4	2.8 (2.5 to 3.1)[b]	2006-2009	4.3 (3.0 to 5.5)[b]	2009-2017	2.3 (2.0 to 2.5)[b]		
**Vermont**	12.5	17.7	41.7	3.2 (3.1 to 3.3)[b]	2006-2011	3.6 (3.3 to 3.8)[b]	2011-2017	2.9 (2.8 to 3.1)[b]		
**Virginia**	12.2	17.0	38.8	3.1 (2.9 to 3.3)[b]	2006-2010	4.1 (3.6 to 4.5)[b]	2010-2017	2.5 (2.4 to 2.7)[b]		
**Washington**	12.2	17.1	39.5	3.2 (3.0 to 3.4)[b]	2006-2013	3.8 (3.5 to 4.1)[b]	2013-2017	2.1 (1.5 to 2.7)[b]		
**West Virginia**	16.4	20.7	26.8	2.2 (1.9 to 2.5)[b]	2006-2009	3.4 (2.1 to 4.8)[b]	2009-2017	1.7 (1.5 to 2.0)[b]		
**Wisconsin**	12.4	17.3	40.0	3.2 (2.9 to 3.4)[b]	2006-2015	3.5 (3.3 to 3.6)[b]	2015-2017	1.7 (0.3 to 3.1)[b]		
**Wyoming**	11.4	16.8	47.1	3.6 (3.5 to 3.8)[b]	2006-2017	3.6 (3.5 to 3.8)[b]				

Source: IQVIA Xponent database.

Abbreviation: AAPC, average annual percent change; 95% CI, 95% confidence interval; Rx, prescription; 95% CI, 95% confidence interval.

[a] Year category presented in each trend represented year groupings as determined by joinpoint regression.

[b] Indicates that the Annual Percent Change (APC) or average APC was significantly different from zero at the alpha = 0.05 level.

eTable 3. Trends in Rate (per 100 Population) of Opioids Prescribed for Duration ≤3 Days, by State, United States, 2006-2017

State	Rx for duration ≤3 days, rate 2006	2017	Change, % 2006-2017	AAPC (95% CI) 2006-2017	Trend 1 Years[a]	APC (95% CI)	Trend 2 Years[a]	APC (95% CI)	Trend 3 Years[a]	APC (95% CI)
United States	16.3	8.9	-45.2	-5.2 (-5.7 to -4.7)[b]	2006-2012	-2.3 (-3.0 to -1.6)[b]	2012-2017	-8.5 (-9.6 to -7.5)[b]		
Alabama	21.1	14.0	-33.6	-3.5 (-4.0 to -3.0)[b]	2006-2012	-0.9 (-1.6 to -0.1)[b]	2012-2017	-6.5 (-7.6 to -5.5)[b]		
Alaska	19.9	11.0	-44.8	-5.4 (-6.2 to -4.6)[b]	2006-2011	-1.4 (-2.9 to 0.2)	2011-2017	-8.7 (-10.0 to -7.3)[b]		
Arizona	19.8	9.7	-51.0	-6.2 (-6.8 to -5.5)[b]	2006-2010	-0.6 (-2.3 to 1.1)	2010-2017	-9.2 (-10.0 to -8.4)[b]		
Arkansas	19.6	13.9	-29.4	-2.9 (-4.1 to -1.7)[b]	2006-2012	1.4 (-0.3 to 3.2)	2012-2017	-7.9 (-10.1 to -5.6)[b]		
California	9.5	4.8	-50.0	-5.9 (-6.8 to -5.0)[b]	2006-2014	-4.6 (-5.3 to -4.0)[b]	2014-2017	-9.1 (-12.4 to -5.7)[b]		
Colorado	19.6	11.0	-44.2	-5.4 (-6.4 to -4.5)[b]	2006-2013	-1.1 (-2.1 to -0.1)[b]	2013-2017	-12.6 (-15.0 to -10.1)[b]		
Connecticut	15.2	10.5	-31.3	-3.0 (-4.2 to -1.8)[b]	2006-2012	-0.5 (-2.3 to 1.3)	2012-2017	-5.9 (-8.3 to -3.4)[b]		
Delaware	18.2	8.6	-52.6	-6.6 (-7.9 to -5.2)[b]	2006-2009	0.6 (-3.1 to 4.4)	2009-2014	-6.4 (-8.7 to -4)[b]	2014-2017	-13.5 (-17.8 to -9.0)[b]
District of Columbia	7.0	5.6	-19.4	-2.3 (-4.8 to 0.3)	2006-2012	4.1 (0.3 to 7.9)[b]	2012-2017	-9.4 (-14.1 to -4.5)[b]		
Florida	15.8	8.2	-48.1	-5.5 (-7.3 to -3.7)[b]	2006-2008	-2.3 (-10.8 to 7.2)	2008-2013	-8.6 (-11.6 to -5.5)[b]	2013-2017	-3.1 (-6.8 to 0.8)
Georgia	20.0	11.2	-44.2	-5.1 (-5.8 to -4.3)[b]	2006-2012	-3.4 (-4.5 to -2.4)[b]	2012-2017	-7.0 (-8.6 to -5.4)[b]		
Hawaii	10.3	7.0	-32.7	-3.5 (-4.4 to -2.6)[b]	2006-2015	-2.2 (-2.6 to -1.7)[b]	2015-2017	-9.5 (-14.6 to -4.0)[b]		
Idaho	21.1	10.9	-48.3	-5.9 (-7.1 to -4.7)[b]	2006-2008	-6.6 (-12.1 to -0.8)[b]	2008-2012	-1.4 (-4.6 to 1.9)[b]	2012-2017	-9.1 (-10.6 to -7.5)[b]
Illinois	15.6	8.3	-46.7	-5.6 (-6.2 to -5.1)[b]	2006-2012	-2.0 (-2.8 to -1.2)[b]	2012-2017	-9.8 (-11.0 to -8.6)[b]		
Indiana	20.5	10.0	-51.0	-6.3 (-6.8 to -5.7)[b]	2006-2012	-4.7 (-5.4 to -3.9)[b]	2012-2017	-8.1 (-9.3 to -7.0)[b]		
Iowa	13.9	9.7	-30.2	-3.0 (-6.0 to 0.2)	2006-2009	-6.5 (-14.4 to 2.1)	2009-2013	5.8 (-3.1 to 15.4)[b]	2013-2017	-8.5 (-13.7 to -2.9)[b]
Kansas	19.0	11.3	-40.8	-4.5 (-5.1 to -3.9)[b]	2006-2013	-2.0 (-2.6 to -1.4)[b]	2013-2017	-8.7 (-10.3 to -7.1)[b]		
Kentucky	25.0	13.1	-47.7	-5.7 (-7.0 to -4.4)[b]	2006-2010	-2.7 (-6 to 0.8)	2010-2017	-7.4 (-9.0 to -5.7)[b]		

State	Rx for duration ≤3 days, rate		Change, %	AAPC (95% CI)	Trend 1		Trend 2		Trend 3	
	2006	2017	2006-2017	2006-2017	Years[a]	APC (95% CI)	Years[a]	APC (95% CI)	Years[a]	APC (95% CI)
Louisiana	27.9	14.9	-46.7	-5.3 (-6 to -4.7)[b]	2006-2012	-3.2 (-4 to -2.3)[b]	2012-2017	-7.9 (-9.1 to -6.5)[b]		
Maine	21.5	8.3	-61.5	-7.6 (-9.5 to -5.7)[b]	2006-2013	-2.8 (-4.6 to -1.0)[b]	2013-2017	-15.4 (-20.3 to -10.3)[b]		
Maryland	13.6	8.4	-38.3	-4.2 (-4.8 to -3.6)[b]	2006-2012	0.3 (-0.6 to 1.1)	2012-2017	-9.3 (-10.5 to -8.1)[b]		
Massachusett s	17.3	7.7	-55.2	-7.1 (-7.9 to -6.2)[b]	2006-2009	0.5 (-1.4 to 2.5)	2009-2015	-7.3 (-8.2 to -6.4)[b]	2015-2017	-16.8 (-21.4 to -12.0)[b]
Michigan	13.3	7.8	-41.1	-4.7 (-5.4 to -4.0)[b]	2006-2012	-1.2 (-2.2 to -0.2)[b]	2012-2017	-8.7 (-10.1 to -7.2)[b]		
Minnesota	14.1	8.7	-38.1	-4.4 (-5.1 to -3.7)[b]	2006-2009	-0.1 (-2.0 to 1.9)	2009-2014	-3.9 (-5.2 to -2.6)[b]	2014-2017	-9.3 (-11.4 to -7.0)[b]
Mississippi	22.5	14.7	-34.4	-4.0 (-5.0 to -3.0)[b]	2006-2014	-1.6 (-2.3 to -0.8)[b]	2014-2017	-10.1 (-13.7 to -6.4)[b]		
Missouri	19.6	10.3	-47.6	-5.5 (-5.9 to -5.0)[b]	2006-2012	-1.5 (-2.2 to -0.9)[b]	2012-2017	-9.9 (-10.9 to -9.0)[b]		
Montana	18.4	10.7	-42.0	-4.6 (-5.2 to -4.0)[b]	2006-2012	-2.4 (-3.2 to -1.5)[b]	2012-2017	-7.2 (-8.5 to -6.0)[b]		
Nebraska	15.7	9.8	-37.6	-3.8 (-4.4 to -3.2)[b]	2006-2012	0.3 (-0.5 to 1.2)	2012-2017	-8.6 (-9.7 to -7.4)[b]		
Nevada	15.0	8.2	-45.3	-5.0 (-5.9 to -4.2)[b]	2006-2012	-3.0 (-4.2 to -1.8)[b]	2012-2017	-7.5 (-9.2 to -5.6)[b]		
New Hampshire	21.7	9.6	-55.6	-7.0 (-8.6 to -5.4)[b]	2006-2012	-2.1 (-3.1 to -1.1)[b]	2012-2015	-7.8 (-13.7 to -1.5)[b]	2015-2017	-19.2 (-25.6 to -12.3)[b]
New Jersey	11.2	6.4	-43.0	-4.8 (-5.7 to -3.9)[b]	2006-2008	1.6 (-2.6 to 6.1)	2008-2015	-3.8 (-4.5 to -3.0)[b]	2015-2017	-14.2 (-18.7 to -9.4)[b]
New Mexico	15.1	10.4	-31.3	-3.4 (-3.9 to -2.8)[b]	2006-2010	2.8 (1.3 to 4.3)[b]	2010-2017	-6.7 (-7.4 to -6.1)[b]		
New York	6.7	5.0	-24.6	-2 (-3.2 to -0.7)[b]	2006-2012	0.8 (-1.0 to 2.7)	2012-2017	-5.2 (-7.7 to -2.7)[b]		
North Carolina	17.5	11.1	-36.5	-3.7 (-4.8 to -2.6)[b]	2006-2013	0.3 (-0.9 to 1.5)	2013-2017	-10.4 (-13.3 to -7.3)[b]		
North Dakota	10.2	6.3	-37.9	-4.5 (-5.2 to -3.8)[b]	2006-2013	0.8 (0.0 to 1.6)[b]	2013-2017	-13.1 (-15.0 to -11.2)[b]		
Ohio	18.4	10.6	-42.3	-4.8 (-5.7 to -3.9)[b]	2006-2010	0.7 (-0.7 to 2.1)	2010-2015	-5.3 (-6.7 to -4.0)[b]	2015-2017	-13.7 (-18.4 to -8.8)[b]
Oklahoma	28.7	11.4	-60.2	-8.4 (-9.1 to -7.7)[b]	2006-2013	-4.4 (-5.1 to -3.7)[b]	2013-2017	-15.0 (-17 to -13.1)[b]		

State	Rx for duration ≤3 days, rate 2006	2017	Change, % 2006-2017	AAPC (95% CI) 2006-2017	Trend 1 Years[a]	APC (95% CI)	Trend 2 Years[a]	APC (95% CI)	Trend 3 Years[a]	APC (95% CI)
Oregon	24.7	12.7	-48.8	-5.6 (-6.4 to -4.8)[b]	2006-2012	-3.2 (-4.3 to -2.1)[b]	2012-2017	-8.4 (-10.1 to -6.7)[b]		
Pennsylvania	12.7	7.5	-40.7	-4.5 (-5.7 to -3.4)[b]	2006-2012	0.4 (-0.3 to 1.2)	2012-2015	-5.8 (-10.2 to -1.1)[b]	2015-2017	-16.4 (-21.0 to -11.4)[b]
Rhode Island	17.2	8.3	-51.7	-5.9 (-7.4 to -4.3)[b]	2006-2012	-2.1 (-4.2 to 0.1)	2012-2017	-10.2 (-13.3 to -6.9)[b]		
South Carolina	24.2	13.0	-46.1	-5.1 (-5.7 to -4.5)[b]	2006-2017	-5.1 (-5.7 to -4.5)[b]				
South Dakota	11.0	9.0	-18.7	-1.4 (-3.2 to 0.4)	2006-2014	1.0 (-0.4 to 2.5)	2014-2017	-7.6 (-13.8 to -0.9)[b]		
Tennessee	33.4	16.2	-51.4	-6.2 (-6.9 to -5.5)[b]	2006-2011	-2.8 (-4.1 to -1.5)[b]	2011-2017	-8.9 (-10.0 to -7.8)[b]		
Texas	16.4	7.8	-52.5	-6.9 (-8 to -5.8)[b]	2006-2012	-2.3 (-3.8 to -0.8)[b]	2012-2017	-12.1 (-14.3 to -9.9)[b]		
Utah	22.9	11.8	-48.5	-5.8 (-6.5 to -5)[b]	2006-2013	-4.2 (-4.9 to -3.4)[b]	2013-2017	-8.6 (-10.6 to -6.5)[b]		
Vermont	14.4	8.2	-43.1	-3.8 (-4.8 to -2.8)[b]	2006-2017	-3.8 (-4.8 to -2.8)[b]				
Virginia	16.6	10.1	-39.5	-3.7 (-4.8 to -2.7)[b]	2006-2012	0.0 (-1.4 to 1.5)	2012-2017	-8.1 (-10.1 to -6.0)[b]		
Washington	23.0	11.6	-49.8	-5.8 (-6.4 to -5.1)[b]	2006-2009	-0.1 (-2.6 to 2.4)	2009-2017	-7.8 (-8.4 to -7.2)[b]		
West Virginia	23.0	10.4	-54.8	-6.9 (-7.7 to -6.2)[b]	2006-2011	-2.0 (-2.6 to -1.3)[b]	2011-2014	-5.8 (-8.8 to -2.6)[b]	2014-2017	-15.7 (-17.4 to -14.0)[b]
Wisconsin	17.2	10.5	-39.1	-4.7 (-5.5 to -3.9)[b]	2006-2011	-1.0 (-2.5 to 0.5)	2011-2017	-7.7 (-8.9 to -6.5)[b]		
Wyoming	23.3	12.2	-47.7	-6.0 (-6.7 to -5.2)[b]	2006-2013	-4.1 (-4.9 to -3.4)[b]	2013-2017	-9.1 (-11.1 to -7.0)[b]		

Source: IQVIA Xponent database.

Abbreviation: AAPC, average annual percent change; Rx, prescription; 95% CI, 95% confidence interval.

[a] Year category presented in each trend represented year groupings as determined by joinpoint regression.

[b] Indicates that the Annual Percent Change (APC) or average APC was significantly different from zero at the alpha = 0.05 level.

eTable 4. Trends in Rate (per 100 Persons) of Opioids Prescribed for Duration ≥30 Days, by State, United States, 2006-2017

	Rx for duration ≥30 days, rate		Change, %	AAPC (95% CI)	Trend 1		Trend 2		Trend 3	
State	2006	2017	2006-2017	2006-2017	Years[a]	APC (95% CI)	Years[a]	APC (95% CI)	Years[a]	APC (95% CI)
**United States**	17.6	24.7	40.2	3.0 (2 to 4.1)[b]	2006-2010	10.3 (8.1 to 12.6)[b]	2010-2015	1.7 (0.0 to 3.5)	2015-2017	-7.1 (-12.2 to -1.7)[b]
**Alabama**	29.3	47.7	62.7	4.7 (3.2 to 6.3)[b]	2006-2009	13.3 (8 to 18.9)[b]	2009-2013	8.1 (3.9 to 12.4)[b]	2013-2017	-4.3 (-6.6 to -1.9)[b]
**Alaska**	15.5	21.1	35.7	3.0 (2.1 to 3.9)[b]	2006-2008	11.7 (6.6 to 17.1)[b]	2008-2015	2.0 (1.3 to 2.7)[b]	2015-2017	-1.8 (-5.7 to 2.3)
**Arizona**	19.6	29.6	51.1	3.8 (3.0 to 4.6)[b]	2006-2011	9.7 (8.6 to 10.8)[b]	2011-2015	1.5 (-0.4 to 3.5)	2015-2017	-5.5 (-9.1 to -1.7)[b]
**Arkansas**	22.9	45.3	97.4	6.4 (3.7 to 9.2)[b]	2006-2008	17.5 (1.0 to 36.8)[b]	2008-2015	5.7 (3.6 to 7.9)[b]	2015-2017	-1.4 (-11.8 to 10.3)
**California**	12.2	17.1	40.3	3.1 (1.9 to 4.3)[b]	2006-2009	11.7 (7.5 to 16.0)[b]	2009-2014	4.0 (1.9 to 6.1)[b]	2014-2017	-6.1 (-9.2 to -3.0)[b]
**Colorado**	13.5	19.7	45.2	3.3 (2.1 to 4.5)[b]	2006-2011	9.2 (7.6 to 10.8)[b]	2011-2015	1.3 (-1.6 to 4.3)	2015-2017	-6.4 (-11.8 to -0.6)[b]
**Connecticut**	14.4	15.9	10.8	0.7 (-1.0 to 2.3)	2006-2012	5.7 (4.4 to 7.0)[b]	2012-2015	-0.6 (-6.8 to 6.1)	2015-2017	-11.4 (-17.5 to -4.9)[b]
**Delaware**	21.9	30.0	36.7	2.6 (-0.1 to 5.4)	2006-2010	8.6 (3.1 to 14.3)[b]	2010-2015	2.6 (-1.9 to 7.2)	2015-2017	-8.1 (-20.5 to 6.3)
**District of Columbia**	5.5	8.1	47.5	2.6 (-0.9 to 6.3)	2006-2017	2.6 (-0.9 to 6.3)				
**Florida**	23.9	29.2	22.1	1.8 (0.9 to 2.8)[b]	2006-2010	9.2 (6.4 to 12.1)[b]	2010-2017	-2.2 (-3.2 to -1.1)[b]	2011-2017	-0.2 (-1.3 to 1.0)
**Georgia**	18.0	28.1	56.2	4.2 (3.3 to 5.1)[b]	2006-2011	9.7 (7.7 to 11.7)[b]	2011-2017	-0.2 (-1.3 to 1.0)		
**Hawaii**	8.2	11.4	38.0	3.1 (2.0 to 4.1)[b]	2006-2010	12.3 (10.5 to 14.2)[b]	2010-2013	0.3 (-4 to 4.8)	2013-2017	-3.5 (-4.9 to -2.1)[b]
**Idaho**	15.4	27.7	79.2	5.4 (4.8 to 6.0)[b]	2006-2011	12.3 (11.5 to 13.2)[b]	2011-2015	3.5 (2.1 to 4.9)[b]	2015-2017	-6.6 (-9.2 to -4.0)[b]
**Illinois**	10.6	19.4	82.3	5.5 (4.3 to 6.8)[b]	2006-2011	9.7 (8 to 11.5)[b]	2011-2015	4.7 (1.6 to 7.8)[b]	2015-2017	-2.5 (-8 to 3.2)
**Indiana**	23.1	33.4	44.6	3.6 (1.1 to 6.1)[b]	2006-2010	12.9 (8.4 to 17.5)[b]	2010-2013	5.4 (-5 to 16.9)	2013-2017	-6.1 (-9.3 to -2.8)[b]
**Iowa**	12.1	19.2	59.0	4.2 (2.7 to 5.6)[b]	2006-2012	9.0 (7.8 to 10.2)[b]	2012-2015	2.4 (-3.3 to 8.3)	2015-2017	-6.7 (-12.0 to -1.0)[b]
**Kansas**	14.3	25.3	77.0	5.1 (3.4 to 6.8)[b]	2006-2012	9.0 (7.6 to 10.5)[b]	2012-2015	3.1 (-3.7 to 10.2)	2015-2017	-3.2 (-9.5 to 3.5)

State	Rx for duration ≥30 days, rate		Change, %	AAPC (95% CI)	Trend 1		Trend 2		Trend 3	
	2006	2017	2006-2017	2006-2017	Years[a]	APC (95% CI)	Years[a]	APC (95% CI)	Years[a]	APC (95% CI)
**Kentucky**	39.6	46.1	16.3	1.2 (0.2 to 2.2)[b]	2006-2011	8.2 (6.1 to 10.4)[b]	2011-2017	-4.3 (-5.6 to -2.9)[b]		
**Louisiana**	22.4	35.0	56.5	3.9 (2.7 to 5.2)[b]	2006-2014	6.2 (5.0 to 7.3)[b]	2014-2017	-1.8 (-6.1 to 2.7)		
**Maine**	21.2	13.1	-38.1	-4.3 (-5.6 to -2.9)[b]	2006-2010	6.1 (3.8 to 8.4)[b]	2010-2015	-5.1 (-7.1 to -3.1)[b]	2015-2017	-20.3 (-26.9 to -13.1)[b]
**Maryland**	14.1	20.2	43.6	3.2 (2.3 to 4.1)[b]	2006-2011	9.3 (8.1 to 10.5)[b]	2011-2015	0.5 (-1.7 to 2.7)	2015-2017	-5.7 (-9.8 to -1.4)[b]
**Massachusetts**	14.7	11.9	-19.0	-2.0 (-2.8 to -1.1)[b]	2006-2012	4.2 (3.5 to 4.8)[b]	2012-2015	-4.6 (-8.0 to -1.2)[b]	2015-2017	-14.8 (-18.3 to -11.1)[b]
**Michigan**	22.4	35.5	58.4	4.2 (3.1 to 5.3)[b]	2006-2011	11.3 (9.7 to 12.8)[b]	2011-2015	2.7 (0.1 to 5.4)[b]	2015-2017	-9.2 (-14.0 to -4.2)[b]
**Minnesota**	9.1	13.0	43.4	3.2 (2.4 to 3.9)[b]	2006-2010	11.5 (10 to 13.1)[b]	2010-2015	1.9 (0.7 to 3.1)[b]	2015-2017	-9 (-12.4 to -5.3)[b]
**Mississippi**	22.5	39.0	73.0	5.0 (3.9 to 6.1)[b]	2006-2010	13.3 (10.9 to 15.7)[b]	2010-2015	3.4 (1.6 to 5.2)[b]	2015-2017	-6.4 (-11.4 to -1.1)[b]
**Missouri**	17.8	29.5	65.3	4.5 (2.4 to 6.6)[b]	2006-2012	8.3 (6.6 to 10.1)[b]	2012-2015	2.2 (-5.9 to 11.1)	2015-2017	-3.3 (-11.0 to 5.1)
**Montana**	19.4	15.6	-19.7	-1.8 (-3.3 to -0.2)[b]	2006-2008	9.8 (0.8 to 19.6)[b]	2008-2012	2.8 (-1.1 to 6.9)	2012-2017	-9.4 (-11.2 to -7.6)[b]
**Nebraska**	11.1	18.0	61.8	4.3 (2.5 to 6.1)[b]	2006-2012	6.8 (5.3 to 8.3)[b]	2012-2015	3.7 (-3.4 to 11.3)	2015-2017	-2 (-8.6 to 5.1)
**Nevada**	28.1	39.5	40.4	3.2 (2.1 to 4.4)[b]	2006-2011	9.0 (6.5 to 11.4)[b]	2011-2017	-1.3 (-2.8 to 0.2)		
**New Hampshire**	12.5	16.0	27.7	2.3 (-0.1 to 4.7)	2006-2011	9.8 (6.9 to 12.7)[b]	2011-2014	1.0 (-8.8 to 11.8)	2014-2017	-8 (-12.9 to -2.8)[b]
**New Jersey**	12.2	18.2	49.0	3.8 (2.9 to 4.7)[b]	2006-2010	9.5 (7.7 to 11.4)[b]	2010-2015	3.3 (1.8 to 4.8)[b]	2015-2017	-5.9 (-10.1 to -1.4)[b]
**New Mexico**	17.5	26.2	50.0	3.8 (2.3 to 5.3)[b]	2006-2010	14.9 (11.7 to 18.2)[b]	2010-2015	0.1 (-2.3 to 2.4)	2015-2017	-7.0 (-14 to 0.5)
**New York**	13.1	17.5	33.9	3.0 (1.4 to 4.7)[b]	2006-2011	9.7 (6.2 to 13.3)[b]	2011-2017	-2.2 (-4.3 to 0.0)		
**North Carolina**	22.1	32.6	47.6	3.8 (2.7 to 4.8)[b]	2006-2013	7.7 (6.4 to 9.0)[b]	2013-2017	-2.8 (-5.2 to -0.2)[b]		
**North Dakota**	11.2	10.6	-6.1	-0.5 (-2.9 to 2)	2006-2008	11.0 (-2 to 25.7)	2008-2015	0.3 (-1.6 to 2.3)	2015-2017	-13.3 (-23.6 to -1.5)[b]

State	Rx for duration ≥30 days, rate 2006	2017	% Change 2006-2017	AAPC (95% CI) 2006-2017	Trend 1 Years[a]	APC (95% CI)	Trend 2 Years[a]	APC (95% CI)	Trend 3 Years[a]	APC (95% CI)
**Ohio**	21.7	27.2	25.5	2.0 (1.1 to 2.9)[b]	2006-2010	11.4 (9.6 to 13.2)[b]	2010-2015	-0.5 (-1.9 to 0.9)	2015-2017	-9.0 (-13.2 to -4.6)[b]
**Oklahoma**	23.4	38.5	64.6	4.7 (3.9 to 5.6)[b]	2006-2013	9.5 (8.4 to 10.6)[b]	2013-2017	-3.1 (-5.1 to -1.1)[b]		
**Oregon**	21.7	19.0	-12.6	-0.9 (-2.4 to 0.6)	2006-2008	13.0 (3.7 to 23.1)[b]	2008-2013	2.9 (0.5 to 5.4)[b]	2013-2017	-11.5 (-13.9 to -9.1)[b]
**Pennsylvania**	17.3	25.4	47.0	3.4 (2.2 to 4.6)[b]	2006-2011	9.1 (7.5 to 10.7)[b]	2011-2015	2.4 (-0.4 to 5.4)	2015-2017	-8.0 (-13.2 to -2.4)[b]
**Rhode Island**	15.1	17.9	18.1	1.5 (-0.1 to 3.1)	2006-2012	6.8 (5.4 to 8.1)[b]	2012-2015	-0.9 (-7.2 to 5.8)	2015-2017	-9.6 (-15.8 to -3.0)[b]
**South Carolina**	22.4	36.1	61.5	4.7 (3.9 to 5.5)[b]	2006-2013	8.8 (7.8 to 9.9)[b]	2013-2017	-2.2 (-4.1 to -0.2)[b]		
**South Dakota**	9.8	14.3	46.0	3.3 (1.9 to 4.8)[b]	2006-2008	11.8 (3.6 to 20.6)[b]	2008-2015	5.0 (3.8 to 6.1)[b]	2015-2017	-9.5 (-15.1 to -3.6)[b]
**Tennessee**	35.8	47.4	32.4	2.6 (2.2 to 3.0)[b]	2006-2010	11.3 (10.5 to 12.1)[b]	2010-2015	0.5 (-0.2 to 1.1)	2015-2017	-8.0 (-10.0 to -6.1)[b]
**Texas**	13.8	20.7	50.5	3.8 (2.7 to 4.9)[b]	2006-2008	12.7 (5.9 to 19.9)[b]	2008-2013	5.5 (3.8 to 7.3)[b]	2013-2017	-2.5 (-4.1 to -0.9)[b]
**Utah**	21.6	26.0	19.9	1.8 (0.6 to 3.1)[b]	2006-2008	10.2 (2.7 to 18.1)[b]	2008-2014	2.1 (0.7 to 3.6)[b]	2014-2017	-3.9 (-6.9 to -0.8)[b]
**Vermont**	11.9	9.9	-16.8	-0.7 (-2.1 to 0.7)	2006-2017	-0.7 (-2.1 to 0.7)				
**Virginia**	14.0	20.5	45.8	3.4 (1.4 to 5.4)[b]	2006-2012	9.3 (7.6 to 11)[b]	2012-2015	0.2 (-7.3 to 8.4)	2015-2017	-8.3 (-15.6 to -0.3)[b]
**Washington**	17.6	17.8	0.9	0.0 (-0.8 to 0.8)	2006-2009	8.1 (5.6 to 10.5)[b]	2009-2015	0.7 (-0.2 to 1.6)	2015-2017	-12.6 (-16.5 to -8.6)[b]
**West Virginia**	46.3	43.4	-6.4	-0.3 (-1.2 to 0.6)	2006-2008	14.7 (8.8 to 20.9)[b]	2008-2014	0.8 (-0.3 to 1.8)	2014-2017	-11.2 (-13.4 to -8.8)[b]
**Wisconsin**	14.2	18.4	29.2	2.2 (0.4 to 4)[b]	2006-2012	7.9 (6.4 to 9.3)[b]	2012-2015	0.8 (-5.9 to 8)	2015-2017	-11.3 (-17.7 to -4.4)[b]
**Wyoming**	15.1	21.9	44.9	3.5 (2.4 to 4.6)[b]	2006-2014	6.3 (5.3 to 7.3)[b]	2014-2017	-3.6 (-7.3 to 0.3)		

Source: IQVIA Xponent database.

Abbreviation: AAPC, average annual percent change; Rx, prescription; 95% CI, 95% confidence interval.

[a] Year category presented in each trend represented year groupings as determined by joinpoint regression.

[b] Indicates that the Annual Percent Change (APC) or average APC was significantly different from zero at the alpha = 0.05 level.

eTable 5. Trends in Rate (per 100 Population) of Opioids Prescribed in High Dosages (≥90 MME per Day), by State, United States, 2006-2017

	High-dosage Rx, rate		Change, %	AAPC (95% CI)	Trend 1		Trend 2		Trend 3	
State	2006	2017	2006-2017	2006-2017	Years[a]	APC (95% CI)	Years[a]	APC (95% CI)	Years[a]	APC (95% CI)
United States	11.5	5.0	-56.7	-7.6 (-8.1 to -7.2)[b]	2006-2009	0.0 (-2.8 to 2.8)	2009-2017	-9.5 (-10.0 to -8.9)[b]		
Alabama	16.3	6.8	-58.5	-7.5 (-9.2 to -5.6)[b]	2006-2017	-7.5 (-9.2 to -5.6)[b]				
Alaska	13.3	8.3	-37.9	-3.3 (-4.4 to -2.2)[b]	2006-2017	-3.3 (-4.4 to -2.2)[b]				
Arizona	11.7	7.5	-35.6	-3.8 (-5.7 to -1.9)[b]	2006-2012	1.1 (-0.1 to 2.4)	2012-2015	-5.6 (-12.7 to 2)	2015-2017	-14.8 (-22.3 to -6.7)[b]
Arkansas	18.4	6.9	-62.6	-9.6 (-11.8 to -7.3)[b]	2006-2017	-9.6 (-11.8 to -7.3)[b]				
California	6.9	3.3	-52.0	-5.7 (-7.6 to -3.6)[b]	2006-2009	2.3 (-5.2 to 10.5)	2009-2017	-8.5 (-10.3 to -6.6)[b]		
Colorado	9.5	4.4	-53.4	-6.7 (-7.7 to -5.6)[b]	2006-2010	2.8 (1.3 to 4.4)[b]	2010-2013	-5.9 (-10.3 to -1.3)[b]	2013-2017	-15.8 (-17.5 to -14.1)[b]
Connecticut	10.5	5.8	-44.3	-5.0 (-6.0 to -4.0)[b]	2006-2013	-1.0 (-2.1 to 0.1)	2013-2017	-11.6 (-14.2 to -8.9)[b]		
Delaware	18.0	9.8	-45.3	-5.3 (-6.9 to -3.7)[b]	2006-2010	10.2 (5.6 to 15)[b]	2010-2017	-13.2 (-15 to -11.3)[b]		
District of Columbia	2.2	1.4	-35.8	-5.6 (-8.9 to -2.3)[b]	2006-2014	0.4 (-2.1 to 2.9)	2014-2017	-20.0 (-30.5 to -7.9)[b]		
Florida	15.2	6.4	-57.7	-7.5 (-9.3 to -5.7)[b]	2006-2010	6.2 (3.7 to 8.7)[b]	2010-2013	-22.3 (-28.5 to -15.7)[b]	2013-2017	-8.2 (-11.4 to -4.9)[b]
Georgia	12.7	5.4	-57.7	-7.6 (-9.0 to -6.1)[b]	2006-2010	-3.6 (-5.3 to -2.0)[b]	2010-2013	-17.8 (-23.3 to -11.9)[b]	2013-2017	-3.2 (-5.6 to -0.7)[b]
Hawaii	6.2	4.6	-25.0	-2.4 (-4.8 to 0.1)	2006-2010	7.0 (3.2 to 11)[b]	2010-2013	-3.3 (-13.3 to 7.8)	2013-2017	-10.3 (-13.8 to -6.6)[b]
Idaho	13.6	6.9	-49.1	-5.5 (-7.0 to -4.1)[b]	2006-2010	-1.4 (-5.1 to 2.4)	2010-2017	-7.8 (-9.6 to -6.0)[b]		
Illinois	9.2	2.8	-70.0	-11.7 (-14.1 to -9.3)[b]	2006-2017	-11.7 (-14.1 to -9.3)[b]				
Indiana	16.3	5.4	-67.1	-9.6 (-12.1 to -7.1)[b]	2006-2009	-2.2 (-11.1 to 7.5)	2009-2017	-12.2 (-14.7 to -9.7)[b]		
Iowa	11.0	3.8	-65.3	-9.7 (-11 to -8.4)[b]	2006-2017	-9.7 (-11 to -8.4)[b]				

State	High-dosage Rx, rate		Change, %	AAPC (95% CI)	Trend 1		Trend 2		Trend 3	
	2006	2017	2006-2017	2006-2017	Years[a]	APC (95% CI)	Years[a]	APC (95% CI)	Years[a]	APC (95% CI)
**Kansas**	13.6	6.8	-49.7	-6.3 (-7.3 to -5.4)[b]	2006-2017	-6.3 (-7.3 to -5.4)[b]				
**Kentucky**	13.2	5.4	-59.3	-8.3 (-8.9 to -7.6)[b]	2006-2010	-0.7 (-2.3 to 1.0)	2010-2017	-12.3 (-13.1 to -11.5)[b]		
**Louisiana**	14.5	5.0	-65.6	-9.1 (-12.5 to -5.6)[b]	2006-2009	-0.9 (-7.4 to 6.1)	2009-2012	-21.6 (-33.8 to -7.2)[b]	2012-2017	-5.7 (-10.1 to -1.0)[b]
**Maine**	15.7	5.7	-63.9	-8.8 (-13.7 to -3.6)[b]	2006-2015	-3.9 (-6.1 to -1.6)[b]	2015-2017	-28.1 (-49.5 to 2.4)		
**Maryland**	9.5	6.3	-34.2	-3.5 (-4.7 to -2.4)[b]	2006-2010	6.8 (3.6 to 10.1)[b]	2010-2017	-9.0 (-10.2 to -7.7)[b]		
**Massachusetts**	8.2	3.8	-54.0	-6.8 (-8.5 to -5.1)[b]	2006-2013	-3.0 (-4.6 to -1.3)[b]	2013-2017	-13.3 (-17.7 to -8.7)[b]		
**Michigan**	12.7	5.4	-57.7	-7.8 (-9.6 to -5.9)[b]	2006-2017	-7.8 (-9.6 to -5.9)[b]				
**Minnesota**	7.7	2.9	-62.4	-8.4 (-9.9 to -6.9)[b]	2006-2009	-2.5 (-7.8 to 3.1)	2009-2017	-10.5 (-12 to -9.1)[b]		
**Mississippi**	15.5	4.9	-68.2	-11.5 (-14.1 to -8.9)[b]	2006-2017	-11.5 (-14.1 to -8.9)[b]				
**Missouri**	16.6	6.2	-62.7	-9.7 (-11.5 to -7.8)[b]	2006-2017	-9.7 (-11.5 to -7.8)[b]				
**Montana**	14.2	5.6	-60.4	-8.0 (-12.7 to -3)[b]	2006-2008	3.6 (-16.2 to 28.1)	2008-2015	-7.5 (-11.1 to -3.8)[b]	2015-2017	-19.6 (-41.6 to 10.7)
**Nebraska**	14.0	4.0	-71.6	-11.9 (-14.0 to -9.7)[b]	2006-2017	-11.9 (-14 to -9.7)[b]				
**Nevada**	13.8	7.5	-45.6	-5.2 (-7.1 to -3.4)[b]	2006-2012	2.8 (0.2 to 5.4)[b]	2012-2017	-14.0 (-17.5 to -10.4)[b]		
**New Hampshire**	13.4	8.0	-40.3	-4.5 (-8.0 to -0.9)[b]	2006-2008	10.8 (-6.5 to 31.3)	2008-2015	-4.8 (-7.5 to -2)[b]	2015-2017	-17.0 (-33 to 2.9)
**New Jersey**	9.8	6.3	-35.7	-3.7 (-4.6 to -2.8)[b]	2006-2010	3.7 (2.2 to 5.3)[b]	2010-2015	-5.7 (-7.2 to -4.3)[b]	2015-2017	-12.5 (-17.4 to -7.3)[b]
**New Mexico**	8.9	4.4	-50.2	-5.0 (-6.8 to -3.2)[b]	2006-2010	6.6 (1.6 to 11.9)[b]	2010-2017	-11.1 (-13.2 to -9)[b]		
**New York**	6.3	4.4	-29.8	-2.5 (-4.1 to -0.8)[b]	2006-2010	1.9 (-2.4 to 6.3)	2010-2017	-4.9 (-6.7 to -3)[b]		
**North Carolina**	14.1	6.0	-57.1	-7.0 (-8.4 to -5.7)[b]	2006-2017	-7.0 (-8.4 to -5.7)[b]				
**North Dakota**	10.8	2.8	-74.3	-11.9 (-13.6 to -10.1)[b]	2006-2017	-11.9 (-13.6 to -10.1)[b]				

State	High-dosage Rx, rate 2006	2017	Change, % 2006-2017	AAPC (95% CI) 2006-2017	Trend 1 Years[a]	APC (95% CI)	Trend 2 Years[a]	APC (95% CI)	Trend 3 Years[a]	APC (95% CI)
**Ohio**	13.8	4.1	-70.0	-10.4 (-12.4 to -8.3)[b]	2006-2009	-1.0 (-8.6 to 7.1)	2009-2017	-13.6 (-15.7 to -11.5)[b]		
**Oklahoma**	19.1	7.9	-58.7	-7.6 (-9.7 to -5.5)[b]	2006-2009	-1.7 (-9.4 to 6.5)	2009-2017	-9.7 (-11.7 to -7.6)[b]		
**Oregon**	13.0	5.9	-54.8	-6.8 (-8.1 to -5.6)[b]	2006-2010	3.3 (1.2 to 5.5)[b]	2010-2014	-7.4 (-10.5 to -4.2)[b]	2014-2017	-18.2 (-21.8 to -14.4)[b]
**Pennsylvania**	12.1	6.7	-44.5	-4.8 (-6.1 to -3.5)[b]	2006-2017	-4.8 (-6.1 to -3.5)[b]				
**Rhode Island**	9.1	4.6	-49.4	-5.6 (-8.2 to -2.9)[b]	2006-2008	6.5 (-7.5 to 22.5)	2008-2012	-3.5 (-10.0 to 3.4)	2012-2017	-11.6 (-15 to -8.1)[b]
**South Carolina**	15.8	6.2	-61.1	-8.8 (-10.6 to -7.0)[b]	2006-2017	-8.8 (-10.6 to -7.0)[b]				
**South Dakota**	11.4	3.7	-67.3	-10.3 (-12.1 to -8.4)[b]	2006-2017	-10.3 (-12.1 to -8.4)[b]				
**Tennessee**	17.6	7.6	-56.7	-7.4 (-9.7 to -5.0)[b]	2006-2009	2.0 (-4.3 to 8.8)	2009-2014	-6.1 (-10.0 to -2.0)[b]	2014-2017	-18 (-25.1 to -10.2)[b]
**Texas**	10.9	2.6	-76.0	-12.2 (-13.7 to -10.7)[b]	2006-2009	-1.5 (-4.2 to 1.2)	2009-2012	-29.1 (-34.3 to -23.5)[b]	2012-2017	-6.8 (-8.9 to -4.6)[b]
**Utah**	12.4	8.4	-32.4	-3.4 (-5.3 to -1.5)[b]	2006-2008	5.8 (-3.4 to 15.9)	2008-2015	-3.8 (-5.3 to -2.2)[b]	2015-2017	-10.7 (-19.9 to -0.5)[b]
**Vermont**	9.0	8.1	-9.9	-0.6 (-2.5 to 1.3)	2006-2013	-2.6 (-4.7 to -0.6)[b]	2013-2017	2.9 (-2.2 to 8.4)		
**Virginia**	10.4	4.9	-53.2	-6.1 (-8.0 to -4.2)[b]	2006-2009	0.8 (-6.3 to 8.5)	2009-2017	-8.6 (-10.3 to -6.8)[b]		
**Washington**	10.6	5.1	-51.8	-6.5 (-7.2 to -5.9)[b]	2006-2010	0.7 (-0.3 to 1.6)	2010-2015	-8.8 (-9.8 to -7.9)[b]	2015-2017	-14.1 (-17.7 to -10.4)[b]
**West Virginia**	14.3	6.0	-58.4	-6.5 (-9.1 to -3.8)[b]	2006-2010	0.8 (-5.8 to 8.0)	2010-2017	-10.5 (-13.8 to -7.1)[b]		
**Wisconsin**	11.0	4.6	-57.7	-7.5 (-9.8 to -5.1)[b]	2006-2010	-1.1 (-4.5 to 2.4)	2010-2015	-6.9 (-10.5 to -3.2)[b]	2015-2017	-20.3 (-31.9 to -6.6)[b]
**Wyoming**	13.0	6.7	-48.6	-5.9 (-7.1 to -4.6)[b]	2006-2017	-5.9 (-7.1 to -4.6)[b]				

Source: IQVIA Xponent database

Abbreviation: AAPC, average annual percent change; MME, morphine milligram equivalents; Rx, prescriptions; 95% CI, 95% confidence interval.

[a] Year category presented in each trend represented year groupings as determined by joinpoint regression.

[b] Indicates that the Annual Percent Change (APC) or average APC was significantly different from zero at the alpha = 0.05 level.

eTable 6. Trends in Rate (per 100 Population) of Opioids Prescribed as Extended-Release or Long-Acting, by State, United States, 2006-2017

	ER/LA Rx, rate		Change, %	AAPC (95% CI)	Trend 1		Trend 2		Trend 3	
**State**	**2006**	**2017**	**2006-2017**	**2006-2017**	**Years[a]**	**APC (95% CI)**	**Years[a]**	**APC (95% CI)**	**Years[a]**	**APC (95% CI)**
**United States**	6.4	5.4	-15.8	-1.5 (-2.4 to -0.7)[b]	2006-2009	5.1 (2.9 to 7.4)[b]	2009-2015	-1.8 (-2.7 to -0.9)[b]	2015-2017	-9.9 (-14.4 to -5.3)[b]
**Alabama**	8.8	8.2	-6.0	-0.7 (-1.4 to 0.0)[b]	2006-2009	7.1 (5.0 to 9.2)[b]	2009-2015	-0.6 (-1.3 to 0.2)	2015-2017	-11.9 (-15.5 to -8.0)[b]
**Alaska**	8.0	7.3	-9.2	-0.8 (-1.5 to 0.0)[b]	2006-2017	-0.8 (-1.5 to 0.0)[b]				
**Arizona**	8.7	7.0	-19.1	-1.9 (-3.3 to -0.4)[b]	2006-2010	4.5 (2.2 to 6.9)[b]	2010-2015	-3.1 (-5.2 to -0.9)[b]	2015-2017	-10.8 (-18.5 to -2.5)[b]
**Arkansas**	6.8	7.2	5.5	0.7 (-1.8 to 3.2)	2006-2008	9.4 (-6.1 to 27.5)	2008-2017	-1.2 (-2.4 to 0.1)		
**California**	4.3	3.5	-19.3	-2.1 (-3.2 to -0.9)[b]	2006-2009	7.2 (3.8 to 10.8)[b]	2009-2014	-2.2 (-4.1 to -0.4)[b]	2014-2017	-10.3 (-13.8 to -6.8)[b]
**Colorado**	6.5	5.4	-16.6	-1.9 (-3.6 to -0.2)[b]	2006-2010	5.3 (2.5 to 8.2)[b]	2010-2015	-3.0 (-5.4 to -0.6)[b]	2015-2017	-12.6 (-21.3 to -2.9)[b]
**Connecticut**	8.0	5.4	-32.6	-3.5 (-4.2 to -2.8)[b]	2006-2008	4.3 (1.1 to 7.7)[b]	2008-2015	-2.3 (-2.8 to -1.8)[b]	2015-2017	-14.4 (-18.1 to -10.6)[b]
**Delaware**	10.2	11.0	8.7	0.8 (-1.3 to 2.9)	2006-2014	4.1 (2.4 to 6.0)[b]	2014-2017	-7.7 (-14.7 to 0.0)[b]		
**District of Columbia**	1.8	1.9	6.0	-0.9 (-4.6 to 3)	2006-2014	4.4 (1.3 to 7.7)[b]	2014-2017	-13.7 (-25.6 to 0.2)		
**Florida**	7.3	6.3	-13.5	-1.0 (-2.1 to 0.1)	2006-2010	4.8 (1.8 to 8.0)[b]	2010-2017	-4.2 (-5.5 to -2.9)[b]		
**Georgia**	6.0	5.3	-10.9	-1.1 (-2.1 to -0.1)[b]	2006-2008	6.7 (1.1 to 12.7)[b]	2008-2014	-1.6 (-2.7 to -0.4)[b]	2014-2017	-5.2 (-8.0 to -2.3)[b]
**Hawaii**	4.9	4.1	-15.2	-1.5 (-2.2 to -0.9)[b]	2006-2010	7.0 (5.8 to 8.2)[b]	2010-2014	-4.0 (-5.5 to -2.4)[b]	2014-2017	-8.9 (-10.9 to -7)[b]
**Idaho**	7.0	7.6	8.9	0.6 (-0.5 to 1.6)	2006-2011	5.6 (4.2 to 6.9)[b]	2011-2015	0.0 (-2.4 to 2.5)[b]	2015-2017	-9.9 (-14.9 to -4.6)[b]
**Illinois**	3.7	3.5	-6.7	-0.6 (-2.9 to 1.8)	2006-2008	6.4 (-5.5 to 19.8)	2008-2015	0.0 (-1.9 to 1.8)	2015-2017	-8.9 (-19.6 to 3.2)
**Indiana**	8.0	6.0	-24.8	-2.5 (-3.5 to -1.5)[b]	2006-2012	3.5 (2.1 to 4.9)[b]	2012-2017	-9.3 (-11.2 to -7.3)[b]		
**Iowa**	5.5	5.0	-9.4	-1.5 (-3.5 to 0.6)	2006-2015	0.8 (-0.3 to 1.9)	2015-2017	-10.9 (-21.8 to 1.5)		

State	ER/LA Rx, rate		Change, %	AAPC (95% CI)	Trend 1		Trend 2		Trend 3	
	2006	2017	2006-2017	2006-2017	Years[a]	APC (95% CI)	Years[a]	APC (95% CI)	Years[a]	APC (95% CI)
**Kansas**	6.9	6.6	-3.1	-0.5 (-1.4 to 0.5)	2006-2008	4.4 (-0.6 to 9.7)	2008-2015	0.7 (-0.1 to 1.5)	2015-2017	-9.0 (-13.5 to -4.3)[b]
**Kentucky**	7.9	5.5	-29.9	-2.7 (-5.3 to -0.1)[b]	2006-2008	7.8 (-4.1 to 21.1)	2008-2011	-0.1 (-10.0 to 10.9)	2011-2017	-7.3 (-9.4 to -5.1)[b]
**Louisiana**	6.6	5.0	-24.0	-2.5 (-3.4 to -1.5)[b]	2006-2017	-2.5 (-3.4 to -1.5)[b]				
**Maine**	10.1	7.7	-23.2	-2.1 (-4.1 to -0.1)[b]	2006-2009	4.5 (-0.6 to 9.9)	2009-2015	-1.0 (-3.0 to 1.1)	2015-2017	-14.5 (-24.6 to -3.1)[b]
**Maryland**	7.0	6.8	-2.2	-0.3 (-1.6 to 1.1)	2006-2010	4.4 (2.0 to 6.9)[b]	2010-2015	-1.1 (-3.2 to 1.0)	2015-2017	-7.1 (-14.1 to 0.3)
**Massachusetts**	6.6	4.4	-33.5	-3.6 (-4.2 to -3.0)[b]	2006-2014	-0.9 (-1.3 to -0.5)[b]	2014-2017	-10.5 (-12.6 to -8.3)[b]		
**Michigan**	6.6	6.6	1.2	-0.1 (-2.2 to 1.9)	2006-2010	7.4 (3.6 to 11.3)[b]	2010-2015	-2.0 (-5 to 1.1)	2015-2017	-9.6 (-19.8 to 2.0)
**Minnesota**	5.4	4.0	-25.0	-2.7 (-4.2 to -1.3)[b]	2006-2010	5.2 (3.0 to 7.5)[b]	2010-2015	-4.4 (-6.4 to -2.3)[b]	2015-2017	-13.3 (-21 to -4.9)[b]
**Mississippi**	6.2	5.9	-5.3	-0.4 (-2.3 to 1.5)	2006-2008	7.1 (-2.8 to 18.1)	2008-2015	-0.3 (-1.8 to 1.2)	2015-2017	-7.7 (-16.6 to 2.2)
**Missouri**	6.9	6.1	-11.0	-1.1 (-2.8 to 0.7)	2006-2008	6.3 (-2.7 to 16.1)	2008-2015	-1.3 (-2.7 to 0.1)	2015-2017	-7.1 (-15.8 to 2.6)
**Montana**	9.7	6.5	-33.0	-3.5 (-4.2 to -2.7)[b]	2006-2008	9.5 (6 to 13.2)[b]	2008-2015	-3.2 (-3.7 to -2.6)[b]	2015-2017	-15.9 (-19.7 to -11.8)[b]
**Nebraska**	5.6	5.4	-4.3	-0.7 (-1.7 to 0.2)	2006-2014	1.0 (0.2 to 1.8)[b]	2014-2017	-5.2 (-8.8 to -1.6)[b]		
**Nevada**	9.2	7.4	-18.8	-1.8 (-2.8 to -0.7)[b]	2006-2008	6.9 (1.7 to 12.4)[b]	2008-2015	-1.8 (-2.6 to -1.0)[b]	2015-2017	-9.7 (-14.9 to -4.1)[b]
**New Hampshire**	10.2	8.2	-19.1	-1.9 (-3.8 to 0.0)	2006-2008	12.2 (2.3 to 23)[b]	2008-2015	-2.2 (-3.6 to -0.9)[b]	2015-2017	-13.2 (-22.4 to -2.8)[b]
**New Jersey**	6.8	5.3	-22.2	-2.2 (-3.8 to -0.5)[b]	2006-2008	2.8 (-4.7 to 11.0)	2008-2015	-1.5 (-2.8 to -0.2)[b]	2015-2017	-9.1 (-17.3 to -0.1)[b]
**New Mexico**	5.7	4.4	-21.3	-2.6 (-5.9 to 0.8)	2006-2010	7.8 (2.5 to 13.4)[b]	2010-2015	-5.2 (-9.5 to -0.6)[b]	2015-2017	-14.9 (-31.6 to 6.0)
**New York**	4.2	4.2	-2.0	-0.1 (-1.3 to 1.1)	2006-2009	7.8 (4.3 to 11.5)[b]	2009-2015	-1.5 (-2.8 to -0.2)[b]	2015-2017	-7.3 (-13.5 to -0.7)[b]
**North Carolina**	7.7	7.1	-8.4	-0.8 (-1.9 to 0.4)	2006-2008	5.6 (-0.3 to 11.9)[b]	2008-2015	0.6 (-0.2 to 1.5)	2015-2017	-11.2 (-16.4 to -5.6)[b]
**North Dakota**	7.6	4.7	-38.2	-4.5 (-6.3 to -2.6)[b]	2006-2008	4.6 (-3.0 to 12.8)[b]	2008-2015	-4.0 (-5.3 to -2.7)[b]	2015-2017	-14.2 (-23.8 to -3.3)[b]

State	ER/LA Rx, rate		Change, %	AAPC (95% CI)	Trend 1		Trend 2		Trend 3	
	2006	2017	2006-2017	2006-2017	Years[a]	APC (95% CI)	Years[a]	APC (95% CI)	Years[a]	APC (95% CI)
**Ohio**	8.2	5.0	-38.4	-4.5 (-6.0 to -3.1)[b]	2006-2010	3.4 (1.5 to 5.4)[b]	2010-2015	-7.5 (-9.4 to -5.5)[b]	2015-2017	-12.1 (-20.3 to -3.0)[b]
**Oklahoma**	8.9	9.0	0.9	0.1 (-3.0 to 3.3)	2006-2008	11.5 (-5.3 to 31.4)	2008-2015	-0.4 (-2.7 to 2.0)	2015-2017	-8.3 (-22.1 to 7.8)
**Oregon**	10.6	6.8	-36.3	-3.7 (-4.9 to -2.4)[b]	2006-2008	11.5 (4.5 to 18.9)[b]	2008-2014	-2.9 (-4.2 to -1.6)[b]	2014-2017	-14.1 (-17.9 to -10.2)[b]
**Pennsylvania**	7.6	6.6	-12.6	-1.3 (-2.1 to -0.5)[b]	2006-2009	5.4 (3.2 to 7.6)[b]	2009-2015	-1.4 (-2.2 to -0.5)[b]	2015-2017	-10.2 (-14.5 to -5.7)[b]
**Rhode Island**	8.1	4.7	-42.3	-4.5 (-6.4 to -2.6)[b]	2006-2008	3.3 (-6.3 to 13.9)	2008-2013	-4.1 (-7.1 to -0.9)[b]	2013-2017	-8.8 (-13.0 to -4.4)[b]
**South Carolina**	6.4	6.3	-0.6	-0.2 (-1.0 to 0.6)	2006-2014	2.1 (1.5 to 2.8)[b]	2014-2017	-6.0 (-8.9 to -3.1)[b]		
**South Dakota**	5.3	4.9	-8.3	-1.1 (-2.8 to 0.7)	2006-2008	7.4 (-1.3 to 17.0)	2008-2015	-0.6 (-2 to 0.7)	2015-2017	-10.2 (-18.3 to -1.4)[b]
**Tennessee**	9.9	8.7	-11.6	-0.7 (-2.3 to 0.9)	2006-2008	8.4 (-1.1 to 18.8)	2008-2014	2.5 (0.7 to 4.2)[b]	2014-2017	-12.0 (-16 to -7.8)[b]
**Texas**	3.4	3.3	-2.3	-0.6 (-1.7 to 0.6)	2006-2008	7.3 (1.4 to 13.6)[b]	2008-2015	-0.6 (-1.4 to 0.3)	2015-2017	-8.1 (-13.5 to -2.3)[b]
**Utah**	9.6	6.9	-27.6	-3.1 (-3.8 to -2.4)[b]	2006-2017	-3.1 (-3.8 to -2.4)[b]				
**Vermont**	7.7	8.3	8.1	1.3 (0.0 to 2.6)[b]	2006-2017	1.3 (0.0 to 2.6)[b]				
**Virginia**	5.2	4.9	-6.7	-0.8 (-2.2 to 0.6)	2006-2010	5.9 (3.3 to 8.5)[b]	2010-2015	-1.5 (-3.6 to 0.6)	2015-2017	-11.5 (-18.7 to -3.6)[b]
**Washington**	9.1	5.9	-35.2	-4.0 (-5.1 to -2.8)[b]	2006-2009	3.2 (0.6 to 6.0)[b]	2009-2015	-4.5 (-5.7 to -3.3)[b]	2015-2017	-12.3 (-19 to -5.1)[b]
**West Virginia**	8.7	6.2	-28.4	-3.0 (-5.0 to -1.0)[b]	2006-2009	6.3 (1.6 to 11.3)[b]	2009-2015	-4.1 (-6 to -2.1)[b]	2015-2017	-12.9 (-23.4 to -0.9)[b]
**Wisconsin**	7.6	5.9	-22.7	-2.4 (-4.5 to -0.3)[b]	2006-2010	4.1 (0.8 to 7.6)[b]	2010-2015	-2.3 (-5.3 to 0.7)	2015-2017	-14.6 (-25 to -2.6)[b]
**Wyoming**	7.7	7.1	-8.9	-0.9 (-2.2 to 0.4)	2006-2014	1.1 (0.1 to 2.2)[b]	2014-2017	-6.2 (-10.8 to -1.3)[b]		

Source: IQVIA Xponent database.

Abbreviation: AAPC, average annual percent change; ER/LA, extended-release/long-acting; MME, morphine milligram equivalents; Rx, prescriptions; 95% CI, 95% confidence interval.

[a] Year category presented in each trend represented year groupings as determined by joinpoint regression.

[b] Indicates that the Annual Percent Change (APC) or average APC was significantly different from zero at the alpha = 0.05 level.

eFigure. (A) Mean Dosage in Morphine Milligram Equivalent (MME) per Day per Pprescription and (B) Mean Duration per Prescription by Formulation in All Ages, by State, United States, 2017.

Data were calculated from IQVIA Xponent database in 2017. Figure 1A: Data markers represent the mean daily dosage (morphine milligram equivalent, MME) per prescription stratified by formulation of prescribed opioids in the United States, 50 states, and the District of Columbia. Figure 1B: Data markers represent the mean duration (days) per prescription stratified by the formulation of prescribed opioids in the United States, 50 states, and the District of Columbia. Blue diamond represents extended release and long acting (ER/LA) formulations; red square represents immediate release (IR) formulations, and black triangle represents all opioids.

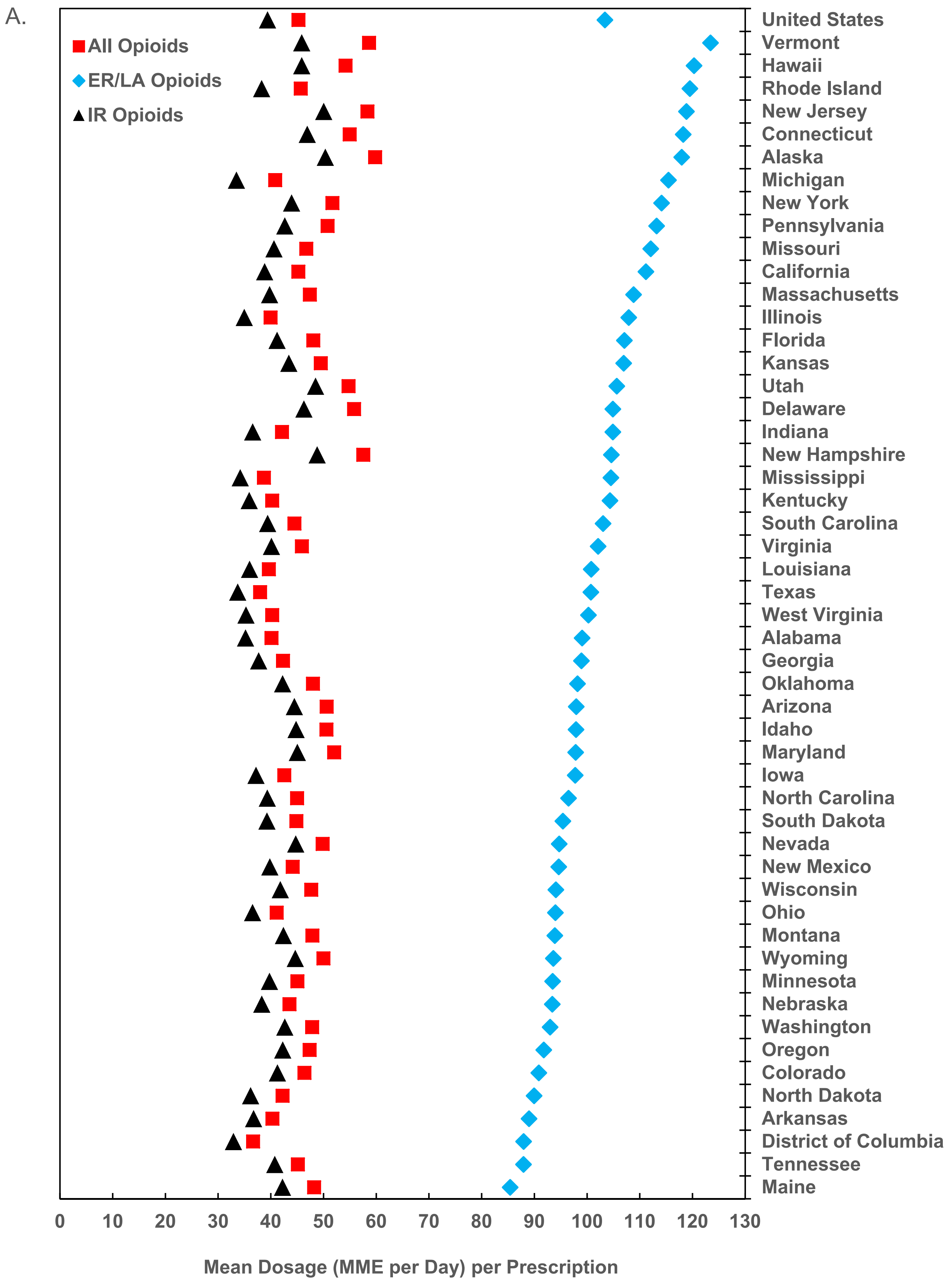
A.
All Opioids
ER/LA Opioids
IR Opioids
United States
Vermont
Hawaii
Rhode Island
New Jersey
Connecticut
Alaska
Michigan
New York
Pennsylvania
Missouri
California
Massachusetts
Illinois
Florida
Kansas
Utah
Delaware
Indiana
New Hampshire
Mississippi
Kentucky
South Carolina
Virginia
Louisiana
Texas
West Virginia
Alabama
Georgia
Oklahoma
Arizona
Idaho
Maryland
Iowa
North Carolina
South Dakota
Nevada
New Mexico
Wisconsin
Ohio
Montana
Wyoming
Minnesota
Nebraska
Washington
Oregon
Colorado
North Dakota
Arkansas
District of Columbia
Tennessee
Maine
0 10 20 30 40 50 60 70 80 90 100 110 120 130
Mean Dosage (MME per Day) per Prescription

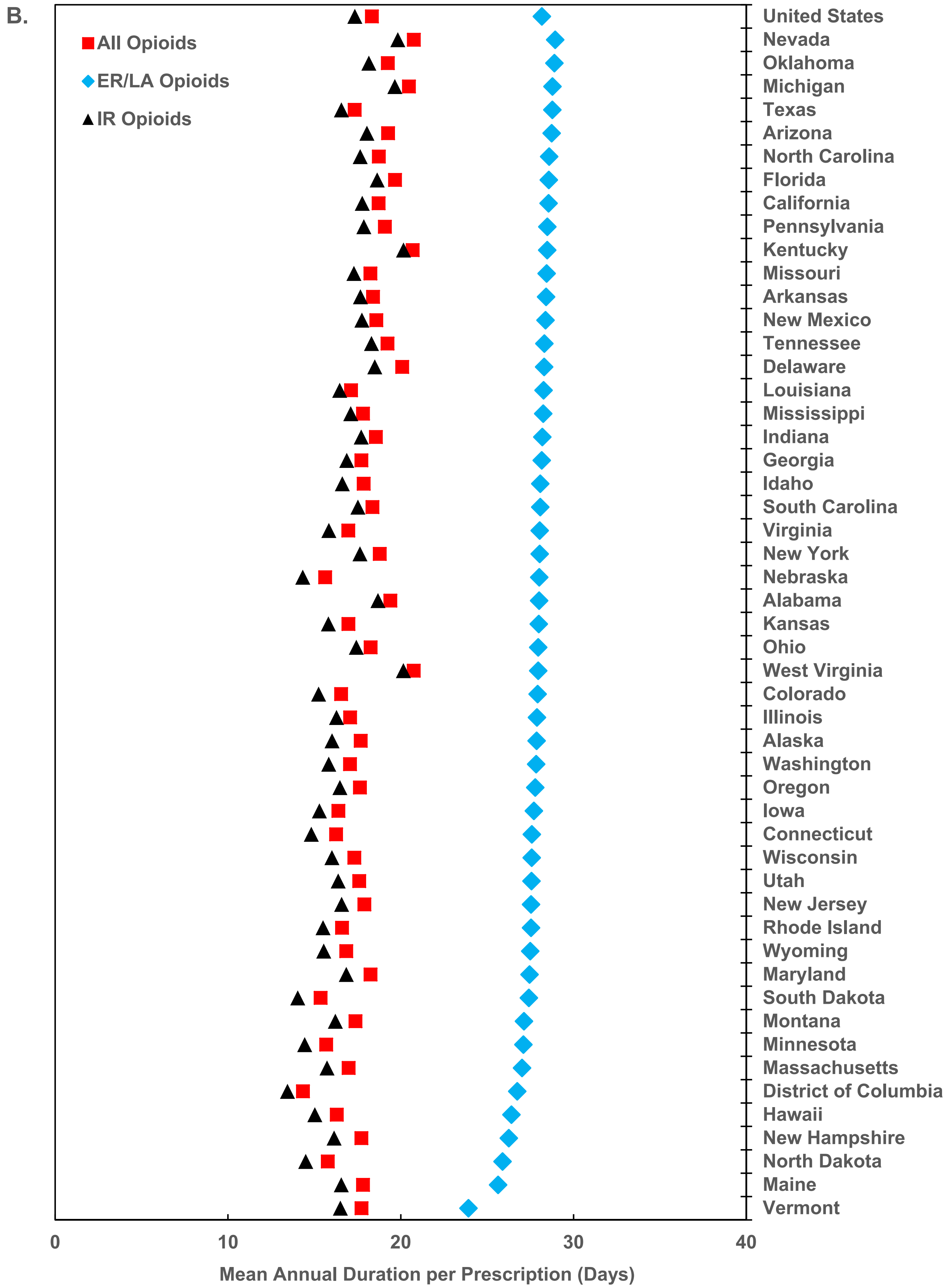

B.
All Opioids
ER/LA Opioids
IR Opioids
United States
Nevada
Oklahoma
Michigan
Texas
Arizona
North Carolina
Florida
California
Pennsylvania
Kentucky
Missouri
Arkansas
New Mexico
Tennessee
Delaware
Louisiana
Mississippi
Indiana
Georgia
Idaho
South Carolina
Virginia
New York
Nebraska
Alabama
Kansas
Ohio
West Virginia
Colorado
Illinois
Alaska
Washington
Oregon
Iowa
Connecticut
Wisconsin
Utah
New Jersey
Rhode Island
Wyoming
Maryland
South Dakota
Montana
Minnesota
Massachusetts
District of Columbia
Hawaii
New Hampshire
North Dakota
Maine
Vermont
0
10
20
30
40
Mean Annual Duration per Prescription (Days)
